# Ancient herpes simplex 1 genomes reveal recent viral structure in Eurasia

**DOI:** 10.1126/sciadv.abo4435

**Published:** 2022-07-27

**Authors:** Meriam Guellil, Lucy van Dorp, Sarah A. Inskip, Jenna M. Dittmar, Lehti Saag, Kristiina Tambets, Ruoyun Hui, Alice Rose, Eugenia D’Atanasio, Aivar Kriiska, Liivi Varul, A. M. H. C. Koekkelkoren, Rimma D. Goldina, Craig Cessford, Anu Solnik, Mait Metspalu, Johannes Krause, Alexander Herbig, John E. Robb, Charlotte J. Houldcroft, Christiana L. Scheib

**Affiliations:** ^1^ Estonian Biocentre, Institute of Genomics, University of Tartu, Riia 23B, Tartu 51010, Estonia.; ^2^ UCL Genetics Institute, Department of Genetics, Evolution, and Environment, University College London, London WC1E 6BT, UK.; ^3^ McDonald Institute for Archaeological Research, University of Cambridge, Cambridge, UK.; ^4^ Department of Archaeology and Ancient History, University of Leicester, University Road, Leicester, LE1 7RH, UK.; ^5^ Department of Archaeology, University of Aberdeen, UK.; ^6^ Alan Turing Institute, 2QR, John Dodson House, 96 Euston Rd., London NW1 2DB, UK.; ^7^ Institute of Molecular Biology and Pathology, CNR, Rome, Italy.; ^8^ Department of Archaeology, Institute of History and Archaeology, University of Tartu, Tartu 51014, Estonia.; ^9^ Archaeological Research Collection, School of Humanities, Tallinn University, Tallinn 10130, Estonia.; ^10^ IDDS Groep bv’s, Gravendijckseweg 37, 2201 CZ Noordwijk, Netherlands.; ^11^ Department History of Udmurtia, Archaeology and Ethnology, Udmurt State University, 1, Universitetskaya St. 1, 426034 Izhevsk, Russia.; ^12^ Cambridge Archaeological Unit, Department of Archaeology, University of Cambridge, Cambridge, UK.; ^13^ Core Facility, Institute of Genomics, University of Tartu, Riia 23B, Tartu 51010 Estonia.; ^14^ Department of Archaeogenetics, Max Planck Institute for the Science of Human History, Jena, Germany.; ^15^ Max Planck Institute for Evolutionary Anthropology, Leipzig, Germany.; ^16^ Department of Archaeology, University of Cambridge, Cambridge, UK.; ^17^ Department of Genetics, University of Cambridge, Downing Street, Cambridge, CB2 3EH, UK.; ^18^ St. John’s College, University of Cambridge, Cambridge, CB2 1TP, UK.

## Abstract

Human herpes simplex virus 1 (HSV-1), a life-long infection spread by oral contact, infects a majority of adults globally. Phylogeographic clustering of sampled diversity into European, pan-Eurasian, and African groups has suggested the virus codiverged with human migrations out of Africa, although a much younger origin has also been proposed. We present three full ancient European HSV-1 genomes and one partial genome, dating from the 3rd to 17th century CE, sequenced to up to 9.5× with paired human genomes up to 10.16×. Considering a dataset of modern and ancient genomes, we apply phylogenetic methods to estimate the age of sampled modern Eurasian HSV-1 diversity to 4.68 (3.87 to 5.65) ka. Extrapolation of estimated rates to a global dataset points to the age of extant sampled HSV-1 as 5.29 (4.60 to 6.12) ka, suggesting HSV-1 lineage replacement coinciding with the late Neolithic period and following Bronze Age migrations.

## Teaser

Medieval human herpes simplex virus 1 genomes implicate Bronze Age migrations in lineage distribution in Eurasia.

## INTRODUCTION

Humans are hosts to a large number of viruses. For many, major uncertainties exist regarding how long they have infected humans, how associated disease pathologies may have changed since their emergence, and the extent to which patterns of viral diversification may mirror the interactions and behaviors of their hosts. Coanalysis of host and pathogen genomes offers rich opportunities to address these questions; however, such studies are often challenged by genomic sampling spanning only relatively recent time scales. When relying on modern data, important assumptions have been made regarding pathogen mutation rates and joint demographic histories, only to be overturned by ancient genomic data (*1*, *2*). Herpes viruses are a prime example of human pathogens for which assumptions have been necessary due to the lack of ancient genomes.

Herpes simplex virus 1 (HSV-1) is a double-stranded DNA virus that affects at least two-thirds of the human population. Most people are infected in infancy or childhood. Most infections are mild or asymptomatic; however, HSV-1 can, in rare cases, lead to severe complications especially in those immunocompromised due to underlying genetic predisposition, infection, or malnutrition (*3*). Following primary infection, the virus becomes latent in sensory neurons (*4*). When triggered by psychological or physiological stress, the virus can reactivate resulting in recurrent labial lesions (*5*, *6*). The emergence of neutralizing antibodies and cytotoxic T cell responses ensures that reactivations typically do not lead to substantial viremia in the immunocompetent host (*7*). A number of human genetic variants have been implicated in susceptibility to HSV-1 in otherwise healthy individuals, covering a range of phenotypes from mild (*8*) to severe, such as the association between *Toll-like receptor 3* deficiency and fatal HSV-1 encephalitis (*9*).

Despite the high global prevalence of HSV-1, there are relatively few genomes available, with modern diversity clearly undersampled (*10*). Comparative studies have indicated that HSV-1 can be clustered into three “phylogroups” associated with geographic origin: I (Europe and America), II (Europe, Asia, and America), and III (Africa) (*11*–*13*). Such a distribution, with phylogroup III falling basal to Eurasian phylogroups, has led to the inference that the origin and subsequent apportionment of genomic diversity in HSV-1 coincided in tandem with human migrations out of Africa (*14*). This codivergence scenario has also been suggested for other agents of common childhood infections (*15*–*17*). Conversely, recent work has suggested that the origin of HSV-1 is far more recent, with little geographic clustering outside of African strains and an estimated time to the most recent common ancestor (TMRCA) dating to less than 7000 years ago (*18*). To date, most of the modern HSV-1 genomes are partial (85%) and/or clinical isolates from highly cosmopolitan centers (*12*, *19*–*21*). The oldest HSV-1 genome (strain HF) was isolated from an individual living in New York in 1925 (*22*) and, like many laboratory strains, has been passaged for generations before being sequenced, which can lead to higher rates of evolution than in natural conditions. Furthermore, recombining viruses, such as HSV-1, may undergo high rates of lineage replacement, meaning that diversity sampled over recent shallow time depths offers limited power to resolve more ancient origins (*23*) while also leading to faster estimates of evolution (*24*). Without direct ancient calibration points, it is difficult to assess the processes giving rise to modern sampled diversity.

Ancient DNA (aDNA) has become an increasingly powerful tool for studying past infections, as next generation sequencing (NGS) has allowed for DNA libraries extracted from skeletal samples to be screened for thousands of microbial species [reviewed by Spyrou *et al.* (*25*)], shedding light on the evolutionary history and phylogeography of past infections. HSV-1 DNA is detectable in blood during primary infection but not during reactivation from latency (*26*); however, herpesvirus DNA and microRNA from nonprimary infections have been recovered from the teeth (*27*) and subgingival plaque (*28*) of living individuals. HSV-1 DNA has also been recovered from the trigeminal ganglia (TG) of cadavers (*6*), indicating that it can reactivate peri- or postmortem. The virus should therefore be common in the archaeological record; however, to date, no full ancient HSV-1 genomes have been published.

## RESULTS

**Fig. 1.**
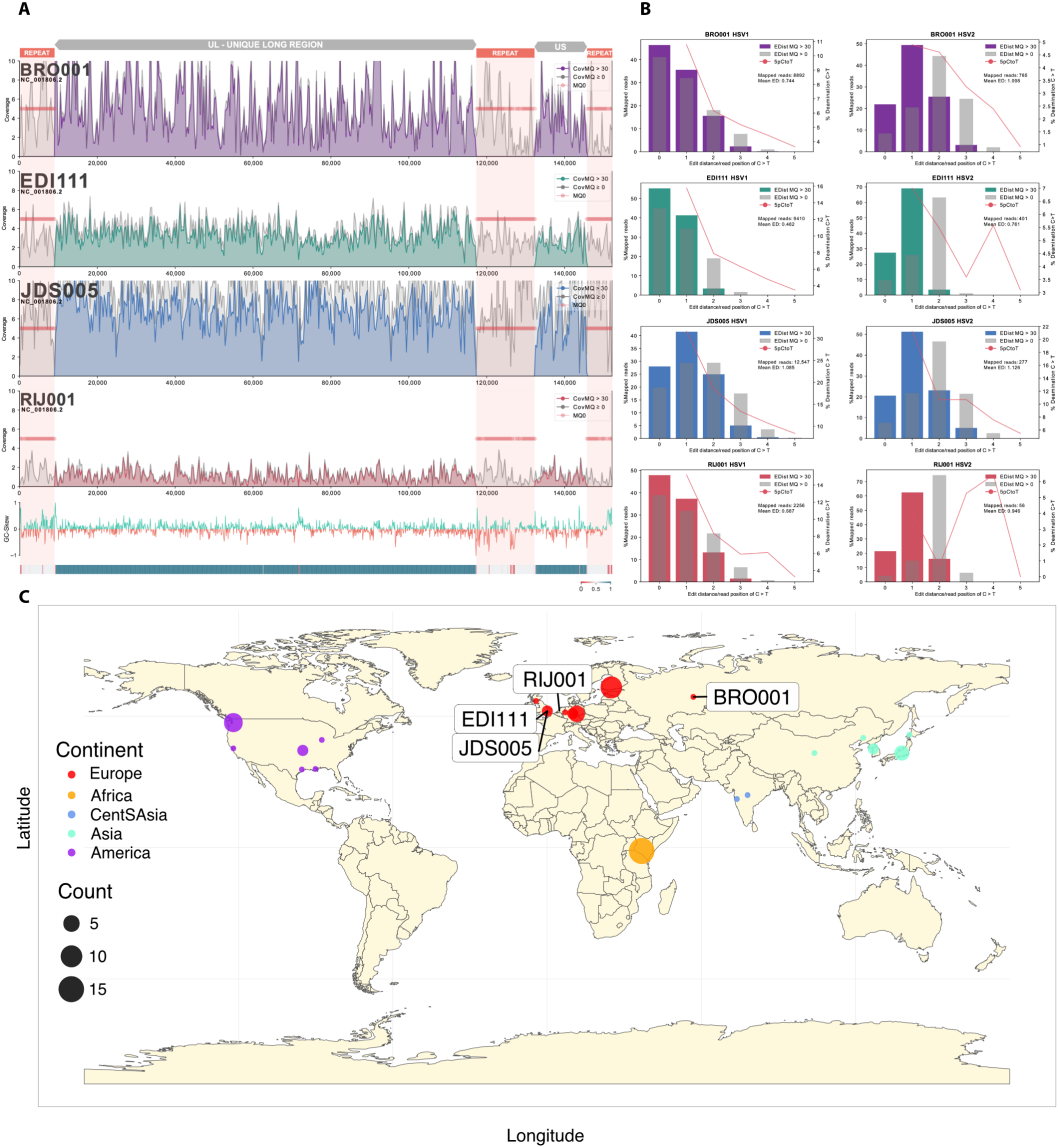
Sequence coverage and edit distance of samples analyzed. (**A**) Linear plots of our mappings to the reference strain 17. The first four plots represent sequence coverage (150-bp windows) and depth across the reference strain for each sample. The last two plots show the GC skew and the mappability estimates based on GenMap (<0.4 in red; >0.6 in blue) (*62*). (**B**) Edit distances for genomes mapped against HSV-1 and HSV-2. Note that JDS005 exhibits more postmortem damage than other genomes and is more divergent from the reference sequence, leading to a higher edit distance from HSV-1. (**C**) World map providing the sampling location of modern HSV-1 accessions jointly analyzed with the three ancient samples: EDI111, JDS005, and BRO001 (as highlighted). Included accessions are provided in data S7. Color provides the continent of sampling and size of the sample count per location as per the legend at left.

We identified four aDNA libraries generated from teeth that contained HSV-1–specific reads. The samples came from a young adult male from an urban medieval hospital cemetery (JDS005; 1350–1450 CE) and an adult female from an early Anglo-Saxon cemetery (EDI111; 500–575 CE) in Cambridgeshire, UK; an adult male from a burial related to the Nevolino culture (BRO001; 253–530 CE) in Russia; and an adult male from the Netherlands (RIJ001; 1600–1700 CE) (Table 1 and the Supplementary Materials). These libraries were then sequenced to higher depth with additional shotgun sequencing or target enrichment to achieve paired human and viral ancient genomes at 0.03 to 11× and 1.2 to 10.16× average coverage, respectively (Fig. 1A and data S1, A and B). In both human and HSV-1 genomes, terminal misincorporation patterns are consistent with aDNA postmortem damage (Fig. 1B and data S1, A and B), and estimated human contamination rates are low (table S1). The RIJ001 alignment was only partial and showed low depth of coverage across the entire reference sequence (Fig. 1A); we therefore excluded the sample from most analyses if not otherwise specified.

**Table 1.** Archaeological summary. Summary of archaeological information for each sample in this study including site-specific numbers, age, date, sex estimation (morphological and genetic), and uniparental (mitochondrial—mtDNA and Y chromosome) genetic typing. All radiocarbon dates are uncalibrated. LLM1, lower left molar 1; PM2, premolar 2; ULM1, upper left molar 1; N/A, not available.

**Sample ID**	**Site**	**Burial no.**	**Skeleton ID**	**Sample taken**	**Morphological age estimate**	**Chronological dating**	**Morphological sex estimate**	**Genetic sex estimate**	**Mitochondrial haplogroup**	**Y chromosome haplogroup**
JDS005	Hospital of St. John	2710	1232	LLM1	Young adult (17–25)	1350–1450 CE*	Male	XY	T1a1	J1a2
EDI111	Barrington A, Edix Hill	38	127A	LLM2	Adult (35–45)	500–575 CE; 1520 ± 27 BP	Female	XX	H1b	N/A
BRO001	Nevolino	N/A	N/A	PM2	Adult	253–530 CE; 1680 ± 35 BP	N/A	XY	U4a1d	R1b
RIJ001	Alphen aan den Rijn	11	16	ULM1	Adult	1600–1700 CE*	Male	XY	H	I2d-CTS2146

*The sample dating is inferred from site stratigraphy using radiocarbon dates of in situ material.

### Retrieved genomes are likely from typical infections

**Fig. 2.**
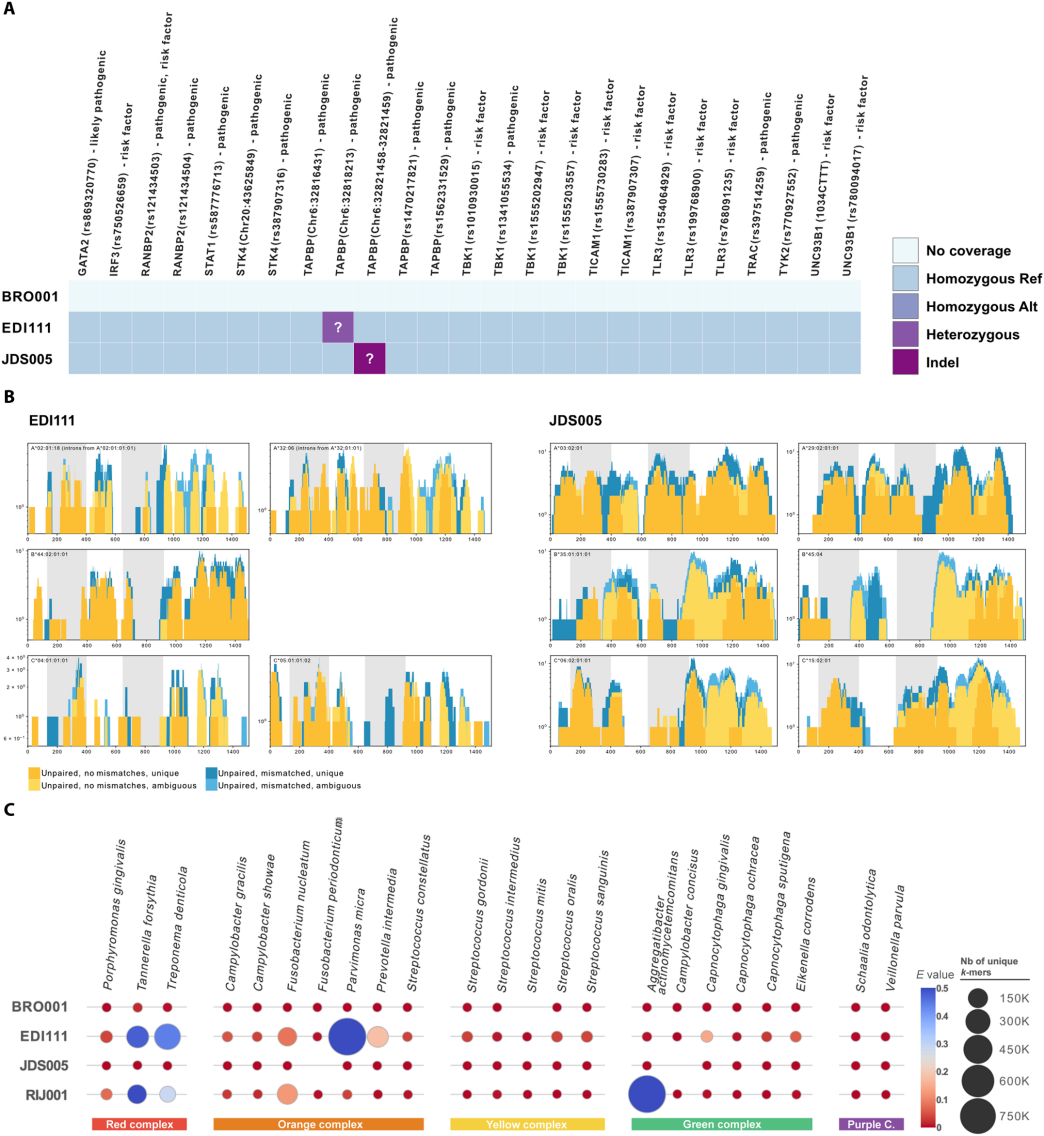
Host susceptibility factors. (**A**) Reference (Ref) versus alternative (Alt) alleles in the host genomes of mutations in genes related to susceptibility to HSV infections as categorized by the ClinVar database downloaded on 17 June 2021. See data S2 for more information. (**B**) Coverage and haplotype assignment of HLA-A, HLA-B, and HLA-C alleles in the two individuals with human genomic coverage of more than three times. Graphs were generated using OptiType (*29*). (**C**) Heatmap showing the unique *k*-mer hits (size) and *E* value (hue) calculated (see Materials and Methods) for all four samples based on KrakenUniq data. Represented are species associated with periodontal disease grouped by complexes based on the work by Socransky *et al.* (*32*) for which hits could be identified for at least one sample. Purple C, purple complex; Nb, number.

Given the scarcity of HSV-1 in the ancient genomics record, we assessed whether these genomes were retrievable due to increased susceptibility of these individuals to infection. First, we looked for potential genetic susceptibility. In the samples with >1× autosomal coverage, we tested for known susceptibility variants and human leukocyte antigen (HLA) alleles implicated in susceptibility to fatal HSV-1 or high runs of homozygosity, which can lead to haploinsufficiency. Host genotypes were called in JDS005 (10.16× average genome-wide coverage) and EDI111 (5.3×); however, neither of these individuals carried variants suggested to cause rare, deadly susceptibility to herpes infections (Fig. 2A and data S2 and S3) nor runs of homozygosity extending more than 4 cM (table S2). We called HLA-A, HLA-B, and HLA-C alleles in both individuals (Fig. 2B) using software designed for NGS data (*29*). Notably, only one HLA-B haplotype was detected in EDI111 (B*44:02:01:01) (Fig. 2B and Table 2). This is unlikely due to a lower coverage as both HLA-A and HLA-C loci had two alleles detected in this individual. Both individuals had alleles common in European populations.

**Table 2.** List of HLA alleles present in JDS005 and EDI111. List of HLA alleles detected using OptiType (*29*) in the samples with more than five times average genome-wide coverage. Bold indicates that the allele is among the top 10 most common alleles in Europe according to the Allele Frequency Net Database (accessed at http://allelefrequencies.net/top10freqsc.asp).

**Sample ID**	**HLA-A**		**HLA-B**		**HLA-C**	
	**Allele 1**	**Allele 2**	**Allele 1**	**Allele 2**	**Allele 1**	**Allele 2**
JDS005	A*03:02:01	A*29:02:01:01	B*35:01:01:01	B*45:04	C*06:02:01:01	C*15:02:01
EDI111	A*02:01:18	A*32:06	B*44:02:01:01	Not detected	C*04:01:01:01	C*05:01:01:02

We then checked for environmental/lifestyle evidence of stress and malnutrition. Bulk isotope data from tooth dentine and bone collagen of JDS005 indicate a diet comparatively low in animal or marine proteins, perhaps due to poverty and/or an inability to take in sufficient nutrition due to other health conditions (Supplementary Materials). No isotopic information is available for the other three individuals. JDS005 also had numerous osteological “nonspecific stress indicators” suggestive of experiencing ill health from a young age, and EDI111 has evidence of arthritis (Supplementary Materials).

HSV-1 has been found at higher prevalence in cases of chronic and aggressive periodontitis (*30*), although its role in pathogenesis is inconclusive (*30*). The skeletons of both EDI111 and JDS005 have dental pathology consistent with periodontal disease, and RIJ001 was a tobacco smoker (Supplementary Materials), which is a major risk factor for periodontitis [reviewed in (*31*)]. Metagenomic screening of our tooth root libraries identified the presence of sequences commonly associated with periodontal pathogens in EDI111 and RIJ001 (Fig. 2C and data S4) including *Tannerella forsythia*, *Treponema denticola*, *Fusobacterium nucleatum*, as well as *Parvimonas micra* and *Aggregatibacter actinomycetemcomitans* for EDI111 and RIJ001, respectively. *T. forsythia* and *T. denticola* form part of the so-called red complex, which has been strongly implicated in periodontal disease (*32*). While these results alone cannot verify the presence of periodontitis in EDI111 and RIJ001, the concurrent presence of multiple periodontal disease–associated bacteria in sufficient amounts to be easily detectable could point toward the presence of an infection as opposed to commensal carriage. While the tested molar root (lower left first molar) from JDS005 did not contain high levels of such bacteria, this individual did suffer from a gross carious lesion in the upper right second molar that resulted in the destruction of >50% of the tooth and an abscess that perforated through the buccal aspect of the right maxilla (affecting the first and second molars). The lower left second molar was also lost antemortem at least several months before death, and this individual experienced chronic maxillary sinusitis (Supplementary Materials). Combined, these strands of evidence point to the likely presence of dental disease in all sampled individuals.

In the absence of known HSV-1 host susceptibility variants, it is not possible to distinguish between primary infection, reactivation of latent virus, and a rare immune-related defect contributing to the detection of HSV-1 in these individuals. No other known pathogenic bloodborne infections (e.g., plague, leprosy, etc.) were detected in any of the tested individuals. Thus, the sum of available evidence indicates that the ancient HSV-1 infections we retrieved were typical, possibly reactivated infections, although these individuals could have had nondetected immunocompromising complications just before death leading to an increased viral load [e.g., sepsis (*33*)]. Our findings highlight the need for more research into the relationship between conditions such as sepsis and HSV-1 reactivation and perimortem HSV-1 reactivation.

We next assessed whether the three full ancient HSV-1 strains carried predicted high consequence genetic mutations/variations, which may lead to higher pathogenicity in the host. On the basis of our single-nucleotide polymorphism (SNP) effect analysis, SNPs within intervals associated with pathogenicity, immunogenicity, or known cellular phenotypes were investigated (data S5 and Supplementary Materials). Of these, the strain BRO001 sampled in Russia carried a mutation leading to a stop-loss in the *US3* domain; however, it is unclear whether this would have affected transcription (*34*). This strain also carried a number of SNPs inferred to have a moderate phenotypic impact in the *UL22/gH* domain (the viral envelope protein), increasing the likelihood that this variation may encompass an antigenic region and thus may be recognized as B or T cell epitopes. No known phenotypically relevant mutations were identified in the Cambridge strains (Supplementary Materials). We do not detect large-scale changes in the genomic composition of ancient when compared to modern HSV-1 strains (data S6).

### Demographic history of HSV-1 in a global context

To place our full ancient HSV-1 genomes into extant diversity, we curated a dataset of modern genome assemblies spanning Europe (United Kingdom, Germany, and Finland), Central and East Asia (China, South Korea, Japan, and India), Africa (Kenya), and the Americas (United States) (data S7 and Fig. 1C). While additional genomes are available from the United States, we subsequently restricted our analysis to those sampled before 1989 to limit the inclusion of highly cosmopolitan strains and/or strains from potentially immunocompromised individuals in our dataset to maximize geographic structure. 1989 was chosen as a cutoff as, from 1989 to 2010, U.S. genomes were increasingly associated with HIV^+^ individuals who were more likely to have HSV coinfections and for whom the data on likely sample/host origin and location of acquisition were less accurate than for earlier samples. We combined the modern dataset with the three ancient higher coverage genomes using a core SNP calling approach and masked hyperdiverse and repeat regions.

**Fig. 3.**
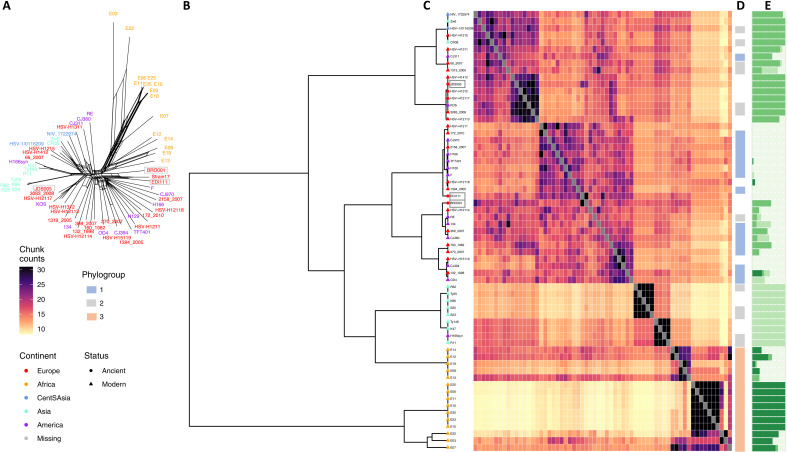
Phylogenetic distribution of HSV-1 genomes. (**A**) SplitsTree neighbor net network of three ancient HSV-1 (highlighted by gray boxes) and curated set of modern global genomes (see table S7). Label colors correspond to the continent of origin (legend at bottom left). (**B**) fineSTRUCTURE hierarchical clustering of all HSV-1 over the core genome alignment. Tips are colored by continent of sampling and ancient/modern status as given by the legend at bottom left. (**C**) Pairwise heatmap, where each pixel provides the average number of haplotype chunks shared between any two considered genomes (*x*/*y* axis) as inferred by Chromopainter, with color scale given at left. (**D**) Colored panel providing the assignment of each sample to phylogroups (where available) previously defined in the literature. (**E**) Unsupervised clustering analysis implemented in ADMIXTURE at *K* = 4 ordered as per fineSTRUCTURE inferred hierarchical clustering.

A phylogenetic network was created over the core alignment of modern and ancient HSV-1 using SplitsTree4 (Fig. 3A) (*35*). The median spanning network recovered major stratification of phylogroups I, II, and III although with high levels of recombination underpinning the clustering. Of note, each of the ancient genomes falls within the diversity observed in Eurasian HSV-1 clusters, broadly defined by phylogroups I and II (*11*), a pattern consistent regardless of the reference genome used (fig. S1). Given the excess of recombination identified in internal nodes of the network, we formally tested for the presence of recombination in the core alignment, detecting a significant correlation between linkage disequilibrium (LD) over genetic distance (*P* = 5.73 × 10^−8^) (fig. S2A). We therefore proceeded to use population genetic methods to identify genetic clusters within our dataset and to characterize the affinity between modern and ancient HSV-1 strains.

Unsupervised model-based clustering (*36*) recovered four well-supported clusters (fig. S2B), again largely delineating the tripartite diversity separating African HSV-1 from European-like “phylogroup I” and Eurasian “phylogroup II,” with our two oldest high coverage genomes sharing inferred ancestry components dominant in European and American strains and with the more recent ancient HSV-1 genome (1350–1450 CE) enriched for an ancestry component identified largely in phylogroup II (Fig. 3, D and E). To further resolve patterns of genetic diversity, we applied a haplotype-based clustering method (*37*), using chromosome painting. Highly consistently, patterns of haplotype sharing partitioned three major groupings with ancient European HSV-1 falling within the two major extant Eurasian clades (Fig. 3, B and C).

As some modern HSV-2 strains have recombined with HSV-1 (*38*, *39*), there is a possibility that HSV-1 strains may, in turn, carry HSV-2 × HSV-1 recombinant regions; however, when assessed using RPD5 (*40*), we found no evidence of recombination with HSV-2 in our ancient strains. We next pruned our alignment for the presence of putative recombinant tracts within HSV-1 strains (fig. S2C and data S8) as their inclusion may violate assumptions of standard phylogenetic approaches. Using the alignment filtered for detectable recombination, we constructed a maximum likelihood phylogeny (fig. S3). The phylogeny largely recapitulated results from recombination-aware analyses and resolved notable substructure within African HSV-1 sampled in Nairobi (*13*), an observation consistent with an African origin. The two oldest ancient genomes fall basal to the diversity of phylogroup I strains, while our medieval genome is placed in phylogroup II, closest genetically to modern strains sampled in Germany and Finland.

**Fig. 4.**
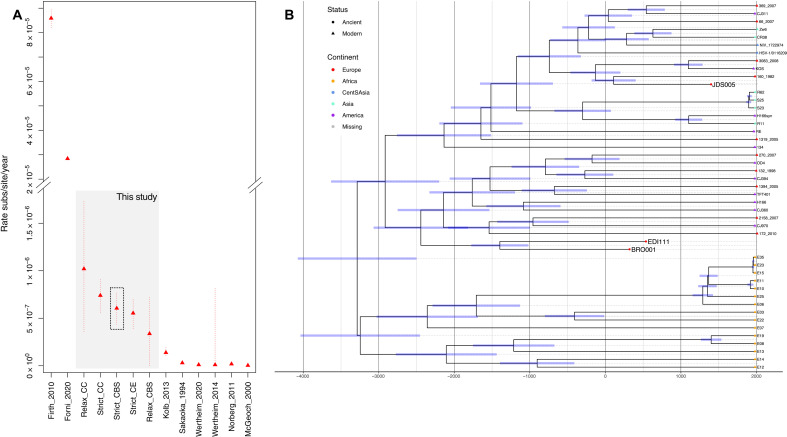
Mutation rates estimated in previous studies of HSV-1 and close relatives and time-calibrated maximum clade credibility tree over a recombination pruned core genome alignment of all HSV-1 genomes. (**A**) *Y* axis provides the rates recovered measured in substitutions per site per year. *X* axis documents eight published studies including five estimates obtained in this study using ancient genomes. Firth *et al.* (*80*) and Forni *et al.* (*18*) provide tip calibration estimates assuming a power law rate decay model. Our most likely model estimate is highlighted in gray, falling between those estimated from modern diversity assuming a power law rate decay and those estimated assuming a scenario of codivergence. Confidence intervals are indicated by dotted lines. (**B**) The time-calibrated phylogeny is inferred following specification of a strict clock model and coalescent skyline population priors assuming a uniform prior bounded by the rates estimated for the Eurasian branch exhibiting temporal signal (fig. S4, A and B). Modern samples are represented by triangular tip points, and ancient samples are depicted with circular tip points. Blue bars provide the 95% HPD interval of the estimated age at each node. Continental origins of the genomes are denoted by the tip color as given in the legend at right.

We assessed whether the extant diversity observed in HSV-1 could be best explained by a deep codivergence with human hosts or instead derived from more recent prehistory. To do so, we tested for the significant accumulation of mutations over the time of sampling in our dataset, taking forward those genomes with reported sample collection dates (data S7). While we were unable to recover a robust global temporal signal, we detected a significant correlation, robust after date randomization resampling, at the node ancestral to all Eurasian samples including our ancient genomes (*R*^2^ = 0.14; *P* = 0.006) (fig. S4, A and B). Using the time of sample collection and radiocarbon estimates for our ancient samples as priors, we estimate the substitution rate and most recent common ancestor of Eurasian diversity, testing six different demographic models and prespecifying relaxed and strict priors on the evolutionary rates (*41*). Models converged to highly similar estimates, with posteriors significantly different from those estimated when sampling from the prior. This resulted in estimated rates of 2.38 × 10^−7^ to 2.49 × 10^−6^ substitutions per site per year and a TMRCA of sampled Eurasian HSV-1 strains spanning 1.87 to 12.73 ka across models, with our highest likelihood implementation supporting a most recent common ancestor (MRCA) of 4.68 (3.78 to 5.65) ka (Fig. 4A and fig. S4, D and E). We estimate the common ancestor of BRO001 and EDI111 to be 3.45 (2.99 to 3.95) ka, with the estimated MRCA of European phylogroup I and pan-Eurasian phylogroup II to approximately the same time periods of 4.50 (3.77 to 5.30) ka and 4.47 (3.64 to 5.33) ka, respectively (Fig. 4B). Two Asian monophyletic clades were placed within the diversity of phylogroup II, dating to 1.32 (1.50 to 1.63) ka and 123.05 years ago (89.67 to 161.70 years ago), respectively. Fixing these estimated rates over the global phylogeny, which includes a further 15 African HSV-1 strains, results in an estimate of extant circulating diversity to 5.29 (4.60 to 6.12) ka [95% highest posterior density (HPD)] (Fig. 4B).

## DISCUSSION

Given the high prevalence of HSV-1 infections in human populations today and the pathophysiology of the virus, HSV-1 should be abundant in archaeological teeth. Since it is a double-stranded DNA virus, it should preserve as well as other double-stranded DNA sources of similar genome size. Our study indicates that the source of the aDNA fragments we retrieved in these individuals was a typical (i.e., life-long and nonfatal) infection. Given both of these factors and the virus’ present-day ubiquity, HSV-1 should be present in more ancient human remains than is currently reflected by available genomic datasets.

Comparing conventional sampling strategies in aDNA, one factor that may have facilitated HSV-1 detection in our samples is that instead of the dental pulp chamber, we targeted the apical root, which is proximal to the TG, where HSV-1 is typically found (*6*). Inter-individual variability in the proximity of apical roots to the nerve canal may be relevant here (*42*). One possibility for the lack of ancient HSV-1 genomes observed to date is that most individuals have a latent infection with low HSV-1 copy numbers in the TG or reduced oral shedding due to a robust immune response (*43*). Today, herpesviruses are more likely to appear in the presence of periodontal disease or other inflammation (*30*), thus HSV-1 might be more likely to be found in ancient individuals suffering the same. Factors that lead to higher oral shedding should be investigated to better inform sampling strategy for ancient HSV-1 studies.

Our results are consistent with either a lineage replacement event or a relatively recent emergence of extant HSV-1 strains. For a lineage replacement to occur, there must be new diversity that becomes more prevalent than the old strains. This could be due to increased transmissibility due to either pathogen or host-specific factors. Since we do not detect large-scale changes in the core genomic composition of ancient and modern HSV-1 strains, it is unlikely due to a change in the pathogen genome; rather, HSV-1 may have become more prevalent over time due to changes in human behavior.

Research has shown that there were migration(s) from the Eastern European steppe region into the rest of Europe (*44*) and likely an increase in population density (*45*) during the Bronze Age. The primary mode of HSV-1 transmission is vertical, from parent to child; however, the addition of lateral transmission as population density increased during the Bronze Age, potentially linked to the introduction of new cultural practices such as the advent of sexual-romantic kissing (Supplementary Materials), may have contributed to a shift in the dominant lineages, which have continued to circulate to this day.

We note that a more recent origin of sampled Eurasian HSV-1 genotypes has support from other studies of modern data (*18*) and in alphaherpesviruses (*46*). For instance, Weinert and colleagues (*46*) dated the emergence of circulating varicella zoster virus diversity to within the last 5000 years and used rates of live attenuated vaccine evolution to suggest that an out-of-Africa scenario was implausible. While varicella zoster virus is spread by infectious aerosols, its substitution rate is expected to be similar to that of other Alphaherpesvirinae (*24*). *Mycobacterium tuberculosis* was also thought to have much older origins and shared coevolutionary history with humans (*47*); however, analyses relaxing the assumptions of codivergence point to a much more recent history (*48*). Similar approaches have been used to estimate conflicting ages of HSV-1 (*14*, *18*). This does not preclude an ancient association of HSV-1 with human hosts. For instance, recent work suggests the presence of putative herpesvirus reads in children dating to 31 ka (*17*). Nonetheless, our results, uniquely aided by observations from these full ancient genomes, suggest that the distribution of extant HSV-1 is more likely the product of more recent events.

The four ancient HSV-1 strains we recover also aid elucidation of the origins of modern strains: For example, the placement of the KOS strain close to the medieval Cambridge and other European sequences suggests Europe as a more likely origin of this strain than Asia as was hypothesized in earlier work (*49*), nor is it likely to be an indigenous American strain (*14*). However, even with the inclusion of ancient genomes, the highly cosmopolitan nature of modern sampling is not ideal for reconstructing historic HSV-1 transmission events, with all estimated TMRCAs highly sampling dependent. Our work therefore highlights the need for more extensive coverage of modern HSV-1, particularly in regions such as Asia and Africa, together with additional observations provided by aDNA samples. Additional ancient genomes, for example, from the Neolithic period, may further revise our understanding of the evolutionary history of this today ubiquitous pathogen and continue to inform on the nature of its association with human hosts.

## MATERIALS AND METHODS

No statistical methods were used to predetermine sample size. The experiments were not randomized, and investigators were not blinded to allocation during experiments and outcome assessment.

### Ethics statement

All skeletal elements were sampled with permissions from the representative bodies/host institutions. Samples were taken and processed to maximize research value and minimize destructive sampling.

### Sampling

Teeth were sampled from skeletons using gloves. Molars were preferred because of having more roots and larger mass, but premolars were also sampled.

### Generation of aDNA libraries

Sampling, decontamination, extraction, purification, and library preparation for JDS005 were carried out as described by Scheib *et al.* (*50*). EDI111 was processed at the Institute of Genomics aDNA facility at the University of Tartu as described by Guellil *et al.* (*51*). BRO001 was processed at the Institute of Ecology and Earth Sciences aDNA facility at the University of Tartu as described by Saag *et al.* (*52*).

### Sequencing

The JDS005 sample was initially shotgun-sequenced on the Illumina NextSeq500/550 using the High-Output single-end 75 cycle kit at the DNA Sequencing Facility in the Department of Biochemistry, University of Cambridge. Once HSV-1 reads were identified in the library using Kraken (*53*), the library was sent to the Max Planck Institute for the Science of Human History, Jena, Germany for 11 additional lanes of sequencing on a HiSeq4000 paired-end 150 cycle kit. Since the libraries were single-indexed and pooled with postcapture libraries for *Mycobacterium leprae*, *Salmonella enterica*, and *Yersinia pestis*, these lanes cannot be used for additional metagenomic searches due to cross-contamination/index hopping from the capture libraries.

EDI111 was shotgun-sequenced on the Illumina NextSeq500/550 using the High-Output single-end 75 cycle kit at the University of Tartu Institute of Genomics Core Facility in three separate runs, the first multiplexed with other samples and the second two alone, followed by three additional runs at the DNA Sequencing Facility in the Department of Biochemistry, University of Cambridge.

BRO001 was shotgun-sequenced on the Illumina NextSeq500/550 using the High-Output single-end 75 cycle kit at the University of Tartu Institute of Genomics Core Facility in a multiplex with other samples. Once HSV-1 reads were identified, the sample was subjected to a custom-designed multiviral capture as described below.

### aDNA authentication

Samtools-1.9 (*54*) option stats and BAMstats-1.25 (http://bamstats.sourceforge.net/) were used to determine the number of final reads, average read length, average coverage, etc. As a result of degradation over time, aDNA can be distinguished from modern DNA by certain characteristics: short fragments and a high frequency of C > T substitutions at the 5′ ends of sequences due to cytosine deamination. The program mapDamage2.0 (*55*) was used to estimate the frequency of 5′ C > T transitions. JDS005 had an average of 20% C > T frequency in the 5′ ends and EDI111 had 12.5%.

To estimate the level of potential contamination in the libraries (table S1), we used the human genome in two ways, the first estimating mitochondrial DNA (mtDNA) contamination and the second the X chromosome in males following the same procedures as detailed in (*51*).

### Metagenomic screening

Generated raw shotgun sequencing data were quality-filtered and trimmed as reported by Guellil *et al.* (*51*). Following these steps, microbial DNA was taxonomically classified using KrakenUniq (*56*) as described by Guellil *et al.* (*51*).

### Targeted capture of HSV-1

Because of low coverage, we enriched the shotgun library of sample BRO001 and RIJ001 for HSV-1 DNA using an Arbor Biosciences Custom MyBaits multispecies viral capture kit (v4), which includes sequences from 283 HSV-1 genome sequences and 261 HSV-2 genome sequences. The captured library was amplified using 2× KAPA HiFi HotStart ReadyMix DNA Polymerase as detailed in (*51*) and sequenced on a NextSeq500 platform (150 bp, paired-end) at the Estonian Biocentre.

### Alignment of viral data to the reference sequence

Raw data were returned in the form of single and paired FASTQ files. Before mapping, single-end datasets were trimmed and filtered as described above for the metagenomic screening. Paired-end datasets were also filtered and trimmed as described in (*51*).

All datasets were merged by library and mapped noncompetitively against the herpes simplex type 1 reference sequences for strain 17 (NC_001806.2), strain S25 (HM585513.2), strain 2158_2007 (LT594106.1), and strain E07 (HM585497.2), to exclude a reference bias, using bwa aln (-n 0.1 -l 1000) and bwa samse (*57*). We then used samtools (*54*) to convert and sort the data to BAM format. Duplicates were marked and removed using Picards’s (*58*) MarkDuplicates module. We then merged sequencing runs for all libraries using samtools merge (-R) and realigned reads around indels using the GATK (Genome Analysis Toolkit) (*59*) RealignerTargetCreator and IndelRealigner modules. We estimated deamination rates using mapDamage2.0 (*55*). Alignments were visualized using a Python script (*60*), and mapping statistics were collected using Qualimap2 (*61*). Edit distances were computed with a custom Python script (*51*). In addition, we also mapped our data noncompetitively to the herpes simplex type 2 strain HG52 reference genome (NC_001798.2) using the same workflow.

All positions were stored using samtools v1.10 mpileup (*-aa* flag) (*54*). Variant positions were then called using bcftools v1.10.2, filtering for sites with a mapping quality of at least 20, a depth of 3, and a quality of 20 and taking forward those positions with an alternate allele fraction >0.8 (MQ > 20, DP > 3, Q > 20, AAF > 0.8). Consensus fasta files were generated using bcftools *consensus* masking those regions of the genome not covered over the reference.

We estimated the mappability across the reference sequence strain 17 using GenMap (-K30 -E2) (*62*). Coverage across gene intervals was calculated using bedtools coverage (*63*), and mean mappability across these intervals was computed using bedtools map. Using SNPEff (*64*), we predicted SNP effects using the National Center for Biotechnology Information (NCBI) reference annotation for strain 17. SNPs with HIGH or MODERATE impact predictions were inspected manually based on filtering criteria (see the Supplementary Materials).

### Genotyping

We additionally performed a genotyping analysis on our alignments to the reference sequence HSV-1 strain 17. Grouping of HSV-1 into three distinct genotypes (A, B, and C) is commonly based on intragenic SNPs in the glycoprotein genes *US4* (*gG*) and *US7* (*gI*) (*65*) and the more recent gene *US2* (*66*). Because of low coverage at some genotyping coordinates, not all SNPs could be assessed in the ancient samples. This led to ambiguous genotypes in genes *US2* and *US4*. Most genotyping loci were covered in our RIJ001 alignment, which was too low coverage for phylogenetic analysis. The sample can probably be typed as (B,C)/B/B. Genotypes are detailed in table S4. However, consistent with previous findings (*11*), the genotyping scheme does not seem to be correlated with the phylogenetic clustering.

### HSV-1 linkage disequilibrium and population genetic analysis

HSV-1 is known to be highly recombinogenic, with recombination acting to decorrelate allele frequencies with characteristic increase with physical distance. To test for the presence of recombination in the core genome alignment, we first thinned core SNPs to exclude those within 80 bp of each other. We then applied TomaHawk (https://github.com/mklarqvist/tomahawk) to estimate the pairwise *r*^2^ between the remaining 812 variant sites, identifying a significant decrease in *r*^2^ with genomic distance (*P* = 5.73 × 10^−8^). To partition the core genetic diversity into clusters, we applied ADMIXTURE v1.3.0 (*67*), first pruning the dataset for sites in high linkage disequilibrium (--indep-pairwise 100 60 0.3) in light of the presence of recombination within the dataset. ADMIXTURE was applied to the resulting alignment encompassing 1231 core SNPs in unsupervised mode for values of *K* ranging from 1 to 15, with the lowest cross-validation error obtained at *K* = 4. Last, a haplotype sharing analysis was applied to recover fine-scale patterns of population structure. Chromopainter v2 (*37*) was run in haploid mode (*-j*) in an all-versus-all manner to “paint” each HSV-1 relative to all others in the dataset. A uniform recombination map was applied assuming a rate of 0.00001 per base pair. Chromopainter was initially run using expectation-maximization to estimate the average switch rate (*-n*) and emission (*-M*) probabilities resulting in mean estimates of *n* = 723.72 and *M* = 0.013, which were fixed in a final run across all individuals (*-a 0 0*). fineSTRUCTURE (*37*) was then applied to cluster individuals based on patterns of haplotype sharing using an estimated normalization parameter *c =* 0.075 running the MCMC (Markov chain Monte Carlo) with 1,000,000 burn-in iterations (*-x*), 2,000,000 sampling iterations (*-y*), retaining every 10,000th sample (*-z*), and applying 1,000,000 tree comparisons during the tree-building step (*-t*).

### Compilation of comparative HSV data

All publicly available assemblies of HSV-1 were downloaded from NCBI by querying NCBIVirus using TaxID 10298 prespecifying a genome length between 150,000 and 200,000. In addition, all accessions not captured by these filters but included in Forni *et al.* (*18*) were included, resulting in a dataset of 299 modern HSV-1 samples (data S7). Within this dataset, accessions were filtered to include only single sample representatives (from studies using longitudinal sampling) and to exclude heavily passaged strains or characterized recombinants (see notes in data S7). The metadata were manually curated to add country and date information through consultation of the dataset prepared by Forni *et al.* (*18*) and through specific requests to associated research groups.

Our interest is in whether regions of the HSV-1 genome can be used for phylogeographic inference, thus two main points were considered in choosing comparative samples: geographic origin and mutation rate. The “natural” mutation rate of HSV-1 in immunocompetent human hosts is lower than in cell culture or immunocompromised individuals (*68*), thus strains from these conditions or studies were left out along with strains sequenced for purposes other than clinical study such as using novel synthetic sequencing methods. Strains isolated from large clinical settings in which the likely geographic origin of the host/strain is not noted were also discarded. An initial analysis considered 162 genomes (including the reference strain 17), which were subsequently filtered to remove those deriving from cosmopolitan centers, for instance, excluding genomes sampled in the United States postdating 1989, which otherwise dominated the dataset. A total of 64 genome sequences were retained for phylogenetic analysis (data S7).

### Preparation of genome sequences

Variants were called for all included modern genomes from their assemblies using the snippy pipeline and specifying contigs as input (https://github.com/tseemann/snippy). All modern and ancient samples were then jointly considered to construct a core genome alignment using snippy-core, as in the work by Lassalle *et al.* (*69*), considering variant positions observed at least 10 times across the dataset and masking difficult to call positions including repeat genes (RL1, RL2, and RS1) together with all regions identified by Szpara *et al.* (*13*) relative to reference strain 17. This resulted in a core SNP alignment including 3815 well-defined variant positions of which each of our ancient genomes (BRO001, EDI111, and JDS005) had 275, 185, and 286 core SNPs, respectively. Across the 63 sample dataset, the distribution of core SNPs ranged from 185 to 803 (mean, 431; 95% confidence interval, 281 to 669). Over the 152,222–bp reference genome, 85,600 positions aligned for BRO001, 112,768 for EDI111, and 118,585 for JDS005 with an average of 119,919 positions over the entire modern and ancient sample dataset. Equivalent analyses were conducted using three representative alternate references to assess the placement of the ancient strains.

### HSV-1 phylogenetic analysis and recombination filtering

A maximum likelihood phylogenetic tree was built over the core genome alignment in IQTree v1.6.12 (*70*) specifying a GTR (General Time Reversible) substitution model run for 1000 bootstrap iterations. To identify sites derived from recent recombination events within the core alignment, we applied 3Seq (*71*) to identify recombination events between all triplet pairs (data S8 and fig. S2C). All identified recombinant blocks were subsequently excluded from the alignment, and a maximum likelihood phylogenetic tree was constructed on the recombination filtered alignment in IQTree as before. Phylogenies were visualized and plotted using ggTree v2.4.1 (*72*). To assess the possibility of recombination between our ancient genomes and HSV-2 strains, we analyzed a full-genome alignment, generated using muscle (*73*), made up of two representative HSV-1 genomes per phylogroup (NC_001806.2, LT594457, MH999845, HM585512, HM585501, and HM585496), a recombinant HSV-1 strain (HM585509), HSV-2 strains (JN561323.2 and KR135308), and our ancient genomes (except RIJ001) using RDP5 (*40*). Our analysis using RDP/GENECOV/Chimera/MaxChi/BootScan/SiScan/3Sec could not detect any recombination event between our ancient genomes and the HSV-2 strains. In addition, we used the query versus reference detection method with our ancient genome set as reference with the same result.

### Phylogenetic dating

We tested for the presence of a significant temporal signal over the recombination filtered core phylogeny, subset to include only those HSV-1 genomes with associated collection dates, using PhyloStems (*74*) and BactDating (*75*). Where a range in sampling date was provided in the associated metadata, we set the date of sampling to the midpoint. While we obtained no global temporal signal basal to the tree, we obtained a significant regression over the ancestral node common to phylogroup I and phylogroup II (fig. S4, A and B) comprising all Eurasian HSV-1 strains. Bayesian tip dating was applied to this subset SNP alignment, specifying the constant site weights, using BEAST v2 (*41*). First, bModelTest (*76*) was applied to estimate the best support site model and associated substitution model, with 74.4% of posterior support for a transversion model (TVM) (fig. S4C). Specifying TVM as prior three possible demographic models (“coalescent constant,” “coalescent exponential,” and “coalescent Bayesian skyline”) were then run in BEAST2 both specifying a strict prior on the clock rate and a relaxed prior on the clock, each time specifying an MCMC chain length of 200 million sampling every 5000th from the run. In each case, convergence was assessed through evaluation of the effective sample size requiring a value of >200 and manual inspection of the MCMC convergence in Tracer v1.7.1. In each case, models were run “without data” by selecting sampling from the prior in the Beauti graphical user interface. Last, to assess model support, each run was repeated with nested sampling (*77*) to generate a marginal likelihood and Bayes factor support for all possible model comparisons. Mean, higher posterior density estimates, and posterior distributions are provided in table S3 and fig. S4D. In addition, an analysis was conducted over the global phylogenetic diversity, specifying a coalescent skyline demographic model and allowing a uniform prior on substitution rates bounded by the estimates obtained for the node common to phylogroup I and phylogroup II. For this analysis, the tree prior was fixed to the maximum likelihood phylogeny estimated over the core, recombination filtered alignment. In all cases, the maximum clade credibility trees were generated using TreeAnnotator v2.6.3, discarding the first 10% of posterior trees as burn-in.

### Alignment of human data to the reference sequence and quality control

The sequence reads were mapped to the human reference sequence (GRCh37/hg19) using the Burrows-Wheeler Aligner (BWA 0.7.12) (*57*) command aln with seeding disabled. After mapping, the sequences were converted to BAM format, and only sequences that mapped to the reference genome were kept using samtools 1.9 (*54*). Next, multiple BAMs from the same individual but different runs were merged using samtools merge, reads with mapping quality under 30 were filtered out, and duplicates were removed with picard 2.12 (http://broadinstitute.github.io/picard/index.html).

### Genetic sex estimation, mtDNA, and Y haplotyping

Genetic sex, mtDNA, and Y chromosome haplogroups were determined as detailed in the Methods section of (*51*).

### Human variant calling and imputation of genotypes

Because of differences in genomic coverage between the samples and generally lower than optimal coverage (>15×) for calling, heterozygous variants were called in three ways: GATK-3.5 -T HaplotypeCaller using the default settings, with ANGSD (*78*) command --doHaploCall, sampling a random base for all genomic positions and pseudo-haploidized by copying the sampled allele, and lastly, by using an imputation pipeline detailed by Hui *et al.* (*79*).

## Supplementary Materials

### This PDF file includes:

Supplementary Materials and Methods Supplementary Text Figs. S1 to S5 Tables S1 to S4 References

## Notes

View/request a protocol for this paper from *Bio-protocol*.
