# Supplementary material for: Ancient herpes simplex 1 genomes reveal recent viral structure in Eurasia
Source: Sci Adv. 2022 Jul 27;8(30):eabo4435. doi: 10.1126/sciadv.abo4435 (PMC9328674; doi:10.1126/sciadv.abo4435)
Supplement: Supplementary file 1 — Supplementary Materials and Methods Supplementary Text Figs. S1 to S5 Tables S1 to S4 References [file sciadv.abo4435_sm.pdf]

Science Advances
AAAS

Supplementary Materials for

Ancient herpes simplex 1 genomes reveal recent viral structure in Eurasia

Meriam Guellil *et al.*

Corresponding author: Christiana L. Scheib, cls83@ut.ee; Charlotte J. Houldcroft, ch504@cam.ac.uk

Sci. Adv. **8**, eabo4435 (2022)
DOI: 10.1126/sciadv.abo4435

The PDF file includes:

Supplementary Materials and Methods
Supplementary Text
Figs. S1 to S5
Tables S1 to S4
Legends for data S1 to S10
References

Other Supplementary Material for this manuscript includes the following:

Data S1 to S9

Materials and Methods

Materials

Craig Cessford, Sarah Inskip, Jenna Dittmar, Aivar Kriiska and A.M.H.C. Koekkelkoren

The Hospital of St. John, Cambridge, UK

The Hospital of St. John, Cambridge was a mediaeval establishment for the care of the poor and infirm, with the exception of pregnant women, lepers, the wounded, crippled and insane(*80*), the assemblage of 400 internments (*c*. 1204-1511)(*81*) included a young adult male (JDS005 / Burial: 198; Skeleton: 1232) aged 17-25 who almost certainly died between 1350-1450 CE. JDS005 is a young adult male individual that has very little evidence of pathology on the skeleton (75-100% complete). There is no evidence of endocranial lesions. Nor is there any evidence of new bone formation on the axial skeleton. This individual presents with both active and healed cribra orbitalia, which may be related to hematopoietic marrow conversion related to anaemia (this remains controversial in the field). There is evidence of dental enamel hypoplasia present on the teeth, which suggest that this individual experienced a 'disruption' in the body's ability to form dental enamel during childhood. This is caused by periods of physiological stress (illness, malnutrition etc.). On the tibiae, there is striated lamellar bone (evidence of a healed periosteal reaction, which is the formation of new bone in response to injury or other stimuli of the periosteum surrounding the bone). This has various possible causes: trauma (unlikely in this case), cancer (unlikely), or things like chronic irritation of the area, or a non-specific infection (more likely).

The skeletal evidence indicates poor dental health. This individual had minor to moderate periodontal disease and most teeth have minor to moderate calculus present along the cemento-enamel junction, with the roots of the anterior teeth also affected. A gross carious lesion (which has resulted in destruction of more than 50% of the tooth) is present on the right second maxillary molar. This alveolus has been destroyed by a large abscess that has perforated through the buccal aspect of the right maxilla (affecting M1 and M2). The left second mandibular molar was lost antemortem. The alveolus is very well healed, which indicates that this tooth was lost at least several months prior to the death of this individual. This individual also experienced chronic maxillary sinusitis. It is possible that this could be related to the observed dental pathology but this is unlikely (because the maxillary sinus was not perforated by the abscess).

Although this individual lived to adulthood, the presence of these 'non-specific stress indicators' suggests that he experienced ill-health early on. These 'indicators' have been linked to increased risk of mortality.

Barrington A, Edix Hill, Cambridgeshire, UK

The site of Barrington A, Edix Hill, Cambridgeshire is an early Ango-Saxon cemetery in use between 500 - 650 CE. EDI111 (PSN650 / grave 38, skeleton 127A), an adult female (35-45 years old), with evidence of arthritis, dental disease and trauma was interred in a double burial, the other inhumation is a 1% complete sub-adult (2-3 years old)(*82*). The lower extremities of the skeleton of 127A were badly damaged by ploughing activity(*82*). The burial was originally assigned to the early phase of the cemetery (500-575 AD), although this phasing is based solely on the presence of amber beads in the burial which is relatively weak

evidence. The radiocarbon determination supports but does not categorically confirm this phasing (Data S9).

Information on the radiocarbon determination for EDI111 is given in Data S9. EDI111 has been radiocarbon dated to 545–590 calCE (1σ interval) or 437–636 calCE (12σ interval). As a lower left 2nd molar was sampled, the radiocarbon determination does not date the death of the individual but when the tooth formed by age 11 - 13. As this individual was osteologically assessed as 35–45 years old when they died, this suggests that the individual died 20 - 30 years after the intervals quoted. Given the date and location of the Edix Hill cemetery it is unlikely that the individuals buried there consumed marine fish, and the δ13C value of -20.4 is compatible with this. Low level consumption of freshwater fish by those buried at Edix Hill is possible, but can not be accurately assessed. Consumption of freshwater fish would have the effect of making the radiocarbon determination older than the death of the individual. The impact of the potential freshwater reservoir effect has not been estimated and is likely to be slight.

Brodovsky, Nevolino, Russia
The Brodovsky burial site is located in the village of Borody in Perm Krai of Russia. On the old riverbank of Shakva several barrows and ground burials have been found(*83*). Archaeological excavations have been carried out there repeatedly since 1898(*83*, *84*). By the burial customs and grave goods, the Brodovsky burial site is related to the Nevolino culture, which existed in Prikamaye from 4th to 9th centuries CE(*84*, *85*). Unfortunately, the analysed tooth comes from a burial, which can no longer be more precisely localised.

Raadhuisstraat 185-193, Alphen aan den Rijn, South-Holland, NL
The individuals buried on the right bank of the river Rhine are most likely victims of a French attack on the villages Bodegraven and Zwammerdam in December 1672(*86*) (pages 65-66). The inhabitants of the villages were massacred and some of the bodies ended up in the Rhine which took them downstream. Some bodies were washed ashore on the excavated area(*86*), which was still unoccupied at that time(*86*) (pages 13-19 and 94-114). A total of four complete individuals were discovered. The bodies were all men from European descent. Two bodies (S36 and S37) were buried disrespectfully together in one grave and face down, which suggests they were French soldiers (i.e. the enemy).

Individual S16 was a male of 26-35 years old(*86*) (pages 57-65). In his youth he probably suffered from an insufficiency of vitamin D, which resulted in a slight bent in the lower legs and a deviation in the ribs. The man was a fervent smoker of clay pipes. Traces of the habit are visible in multiple places on the teeth, where the hard clay pipe, usually put in the same place in the mouth, has worn the teeth (see fig. S1).

Methods
Functional analysis of ancient HSV-1
Charlotte Houldcroft and Meriam Guellil

Variant annotation
We annotated our vcf files using snpEff(*64*) with a custom database for the HSV-1 reference strain based on the gff3 for NC_001806.2. For the annotation, we also included indels in our vcfs, which were inspected by eye to avoid misalignments and misidentifications. Using

bedtools(*63*) and our GenMap data we further computed the number of SNPs per gene and the coverage in gene intervals (>MQ30). Variants are listed in Data S5.

We investigated moderate and high impact SNPs within glycoprotein intervals associated with an immune response based on the following criteria: effect grade, coverage and depth of coverage of the interval of interest, MQ>=30 at the position and DP>=3 at the position.

Selected SNPs:
JDS005

UL27: gB - Two SNPs predicted to be moderate but they do not fall into Uniprot-predicted features, such as the virion surface. Unlikely to be significant.

UL22: gH - Four non-synonymous SNPs (p.Ala150Thr; p.Ser138Ala; p.Thr127Ile; p.Pro110Ser) are all in the Uniprot-predicted virion surface portion of the glycoprotein, increasing the chances that these variants may be recognised as epitopes by the immune system(*87*). Glycoprotein gH also has a role in cell infection (fusion of the virus for entry)(*88*).

US4: gG - This is a short protein, so it is hard to predict the effect of a non-synonymous SNP at aa3.

US6: gD - This SNP is not in the transmembrane domain of gD, but lies just outside the N terminus (amino acids 1-29), which are recognised; speculated to be neutral(*89*).

EDI111

UL27: gB - One moderate effect SNP, but AA873 is an intravirion (predicted) domain, so less likely to be an epitope.

BRO001

US3: - One high effect (stop_loss) SNP in this region, which is a significant virulence factor for HSV-1. This region encodes serine/threonine kinase.

UL27: gB - One moderate effect SNP. As in EDI111, it lies within an intravirion (predicted) domain, so less likely to be an epitope.

UL22: gH - A number of predicted moderate SNPs are in the Uniprot-predicted virion surface portion of the glycoprotein, increasing the chances that these variants may be recognised as epitopes by the immune system.

US4: gG - This is a short protein, so it is hard to predict the effect of a non-synonymous SNP at aa3 (same as the JDS genome, thus it may be a common polymorphism).

US6: gD - Just outside the predicted transmembrane domain of gD; speculated to be neutral.

Host Susceptibility analyses
The ancient human vcf files were annotated with the latest ClinVar vcf downloaded from the ftp site on 27/01/2020. The resulting annotated snps were filtered for clinical significance “pathogenic”, “likely pathogenic” and for “herpes” information tags (Data S3). For JDS005 (~11X) variants with DP ≤ 3 were discarded; however, since EDI111 had a much lower coverage of ~6X, all variants with DP ≥ 2 were kept and evaluated by hand. Variants called

homozygous were accepted. Due to aDNA damage present and average genomic coverage below 15X, variants called heterozygous were assessed as either likely heterozygous (ratio of ref:alt at ~1:1) or likely erroneous (ratio of ref:alt >2:1 and a C > T or G > A transition). Interestingly, though not related to HSV-1, JDS005 carries an autosomal dominant variant for nocturnal frontal lobe epilepsy (rs12721510, Data S3), which may explain why he was in the Hospital of St John the Evangelist at a young age.

In addition, specific genomic regions of interest for susceptibility to Herpes viruses were identified by searching ClinVar for "Herpes" and including all genes in which pathogenic SNPS were listed. This included 14 genes. A bed file of gene positions was used to extract the haplotypes from a merged VCF file using bcftools (*90*).

Herpesviridae have been found to be more abundant in cases of chronic and aggressive periodontitis(*28*, *91*) though the connection with HSV-1 remains inconclusive(*30*, *92*). Sequencing reads from EDI111 point to the presence of bacteria commonly associated with minor to moderate periodontal disease along with a number of other microorganisms that may indicate poor dental health.

Isotope Analysis

Analysis of carbon and nitrogen isotopes in archaeological human dentine and bone collagen can be used to provide information about diet in the past(*93*, *94*). This is based on the principle that carbon and nitrogen isotope values in skeletal tissues largely reflect the isotopic composition of the diet consumed during life(*95*, *96*) and these signatures are preserved in archaeological skeletal remains. Broadly, carbon isotope values can provide information about the types of plants consumed (C_3 vs C_4) (*97*, *98*), nitrogen isotope values can provide information about animal protein consumption and subsequent trophic level(*93*, *99*, *100*) and analysis of a combination of carbon and nitrogen isotope values can provide information about marine fish consumption(*99*, *101*, *102*). In addition, analysing multiple skeletal tissues from an individual can provide information about diet across an individual's lifespan(*103*). As primary dentine does not remodel(*104*), analysis of carbon and nitrogen isotopes in dentine is representative of diet at the time of formation in childhood, whereas bone constantly remodels throughout life, at varying rates(*105*) and therefore isotope values in bone collagen represent diet in the years before death(*93*).

Generation of isotopic data

Dentine collagen from the root (enamel-dentine junction to root apex) of an upper left 2nd molar (representing diet from approx.8.5-12.5yr(*106*) from JDS005 was analysed, and combined with collagen isotope data generated by Price (2013)(*107*) from a rib midshaft (representing diet in the years before death(*105*)). EDI111 and BRO001 were not analysed as they were outside the scope of the After the Plague project.

Preparation of collagen samples was carried out in the Dorothy Garrod Laboratory, McDonald Institute, University of Cambridge, following the laboratory standard operating procedures based on a modified version of the Longin method (Longin 1971, Collins and Galley 1998, Richards and Hedges 1999). An approx. 300mg sample of root dentine and a 500-1000mg sample of rib bone were cut using a hand-held Dremel drill with a diamond-tipped cutting wheel. The sample surfaces were then abraded using a sandblaster to remove surface contaminants. To extract the collagen, samples were first demineralized in approximately 8ml of cold 0.5M aq. hydrochloric acid (HCl), then rinsed with distilled water

and heated in approximately 8ml of pH 3.0 H2O for 48hrs at 75°C until gelatinized. The samples were then filtered using Ezee filters (60-90μm), frozen (-20°C, then -80°C) and placed into a freeze-drier until fully lyophilized. Analysis of the collagen samples was carried out in the Godwin Laboratory, Department of Earth Sciences, Cambridge. All samples were analysed in triplicate. For each one of the triplicates, 0.8mg (±0.1mg) of lyophilised collagen was weighed into a tin capsule. For each batch of samples submitted for analysis, a suite of in-house standards were also weighed into tin capsules and analysed (caffeine, alanine, nylon, protein, EMC). Analysis was carried out using a Costech automated elemental analyser coupled with a Thermo Finnigan MAT253 isotope ratio mass spectrometer in continuous flow mode. All samples are reported on the international scale relative to VPDB for carbon and AIR for nitrogen (Hoefs 1997). Based on replicate analyses of standards, analytical error was <±0.2‰. All results were quality checked to see if they were within the acceptable atomic C:N ratio range of 2.9-3.6 (DeNiro 1985) and above the minimum acceptable %C and %N threshold (>4-5% for C and >13% for N (Ambrose 1990)).

Results

Both tissues from JDS005 provided successful $\delta^{13}C$ and $\delta^{15}N$ values: dentine collagen $\delta^{13}C$ = -19.5‰ and $\delta^{15}N$ = 9.9‰, rib collagen $\delta^{13}C$ = -19.5‰ and $\delta^{15}N$ = 9.8‰. The C:N ratios were within the acceptable range of 2.9-3.6(*108*) and the %C and %N in the samples were above the minimum thresholds (taken as >13% for C and >4-5% for N(*109*).

The isotope values for JDS005 are below the mean for the general adult population from the Hospital of St John (mean dentine collagen $\delta^{13}C$ = -19.2‰, $\delta^{15}N$ = 12.1‰, n=55; mean rib collagen $\delta^{13}C$ = -19.0‰, $\delta^{15}N$ = 12.5‰, n=114)(*110*). More specifically, they are below the mean for adult males in the Hospital (mean dentine collagen $\delta^{13}C$ = -19.1‰, $\delta^{15}N$ = 12.1‰, n=31; mean rib collagen $\delta^{13}C$ = -18.9‰, $\delta^{15}N$ = 12.6‰, n=64) and general Parish adult males from the nearby High and Late Medieval site of All Saints by the Castle (mean dentine collagen $\delta^{13}C$ = -19.5‰, $\delta^{15}N$ = 11.9‰, n=14; mean rib collagen $\delta^{13}C$ = -19.3‰, $\delta^{15}N$ = 12.5‰, n=25) (ibid.). Indeed, JDS005 has some of the lowest $\delta^{15}N$ values observed out of all the High and Late Medieval adults sampled from the Cambridgeshire population (ibid.).

JDS005 is also unusual in the lack of meaningful change in the isotope values between their dentine and rib collagen (difference in values is below the analytical error of ±0.2‰). This is different to the rest of the adult population from the Hospital of St John, who generally exhibited a change in values between the tissues, particularly in $\delta^{15}N$ (mean $\Delta^{13}C_{\text{rib coll-dentine coll}}$ = 0.1‰, mean $\Delta^{15}N_{\text{rib coll-dentine coll}}$ = 0.5‰, n=53). JDS005 is well below the mean $\Delta^{15}N_{\text{rib coll-dentine coll}}$ values for adult males in the Hospital (0.7‰, n=30).

Overall, the isotope values for JDS005 are indicative of a C_3 plant based diet, with little regular terrestrial animal protein input and little to no marine protein input. The lack of change in isotope values between the dentine and rib collagen indicates no detectable change in diet between childhood and adulthood. In terms of $\delta^{15}N$ values, JDS005 appears to be quite unusual compared to the rest of the population buried in the Hospital of St John, as well as the Cambridge town population as a whole. Their much lower values and lack of change could indicate they were consistently consuming less animal proteins than much of the rest of the population of the town. When taken in context with their young age at death, pathologies present, short stature and eventual burial in the Hospital, this may be taken as an indication of someone who could not afford regular animal or marine proteins in their diet, someone who was too sickly to consume them, or even someone who chose not to consume them.

Supplementary Text

A brief history of kissing

Christiana Scheib

While there are Neolithic figurines that have been interpreted as a couple embracing(*111*), the earliest known written record of kissing is a Bronze Age manuscript from South Asia (*112*) (Fig. 4B). The custom may have made its way back to the Mediterranean with the return of Alexander the Great's troops around 300 BCE. Three hundred years later, the Emperor Tiberius is said to have tried to ban kissing at official functions in an effort to stop the spread of disease (unclear whether the disease was herpes)(*113*).

Such a transition in transmission routes would allow for transmission within larger host communities as well as at the intersection of populations when they meet. Sexual-romantic kissing is far from universal in human cultures (and is also observed in Chimpanzees and Bonobos), but is higher in prevalence in modern European, Middle Eastern and Asian cultures(*114*).

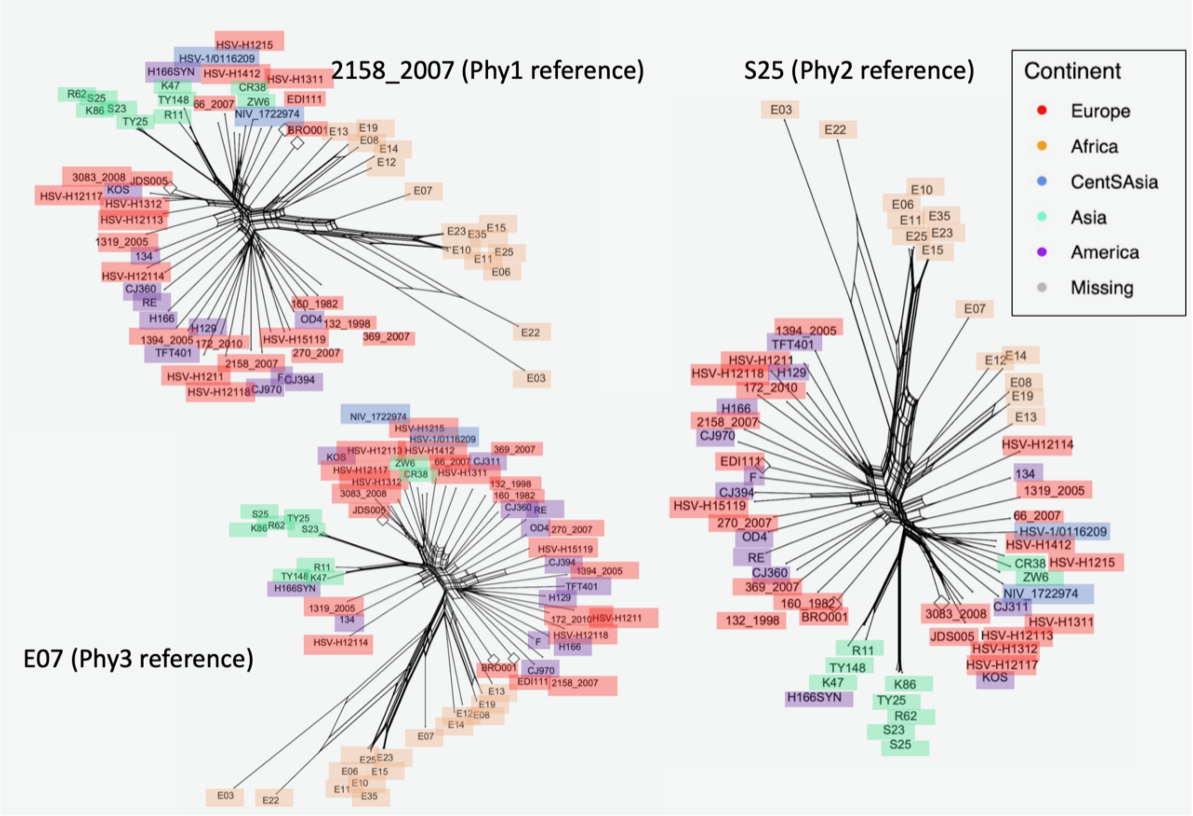

Fig. S1. SplitsTree neighbour nets for core genome alignments obtained when using three distinct reference genomes. (**A**), 2158_2007, (**B**), S25, (**C**), E07. Ancient samples are highlighted with a diamond with accession names as per Data S7. Samples are highlighted by colour according to region.

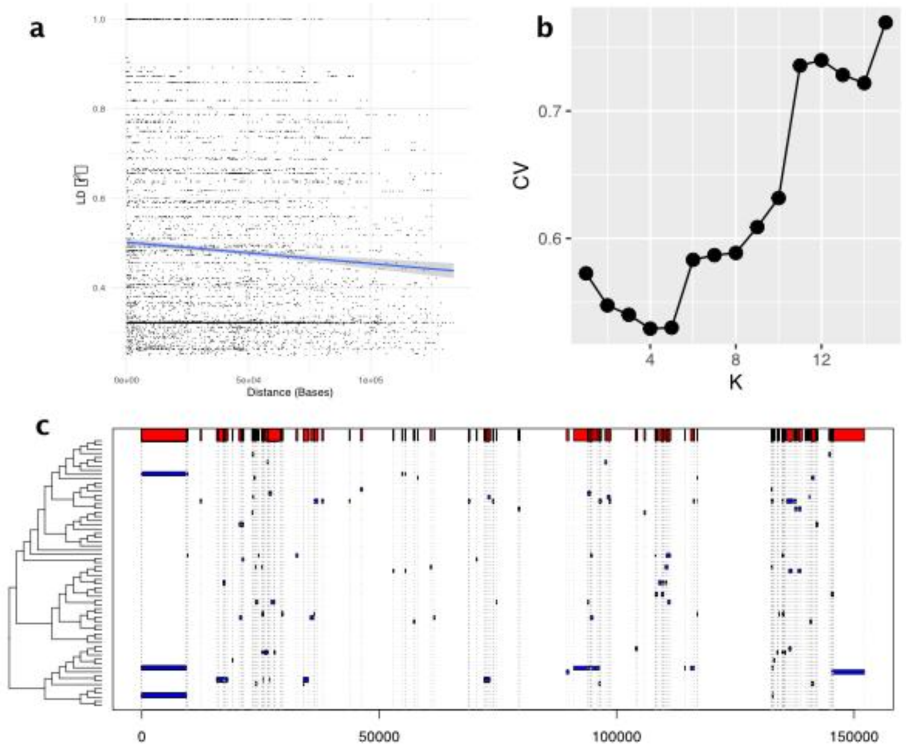

Fig. S2. LD vs. genetic distance, CV for ADMIXTURE and Recombination regions
(**A**), Decline in LD (r 2 , y-axis) over genetic distance (x-axis) specifying a minimum r2 value of 0.25. to aid visibility. Following linear regression R^2 0.1, p-value 0.004. Confidence interval indicated by grey shaded area. (**B**), ADMIXTURE cross-validation estimates following application of unsupervised clustering. Inferred ancestry components at K=5 are provided in Figure 3c. (**C**), Putatively recombinant sites identified by 3Seq. Blue provides individual recombination blocks identified in triplet parent child combinations (see Data S8). Red panel at top provides the combined recombinant tracts identified over the HSV-1 reference genome.

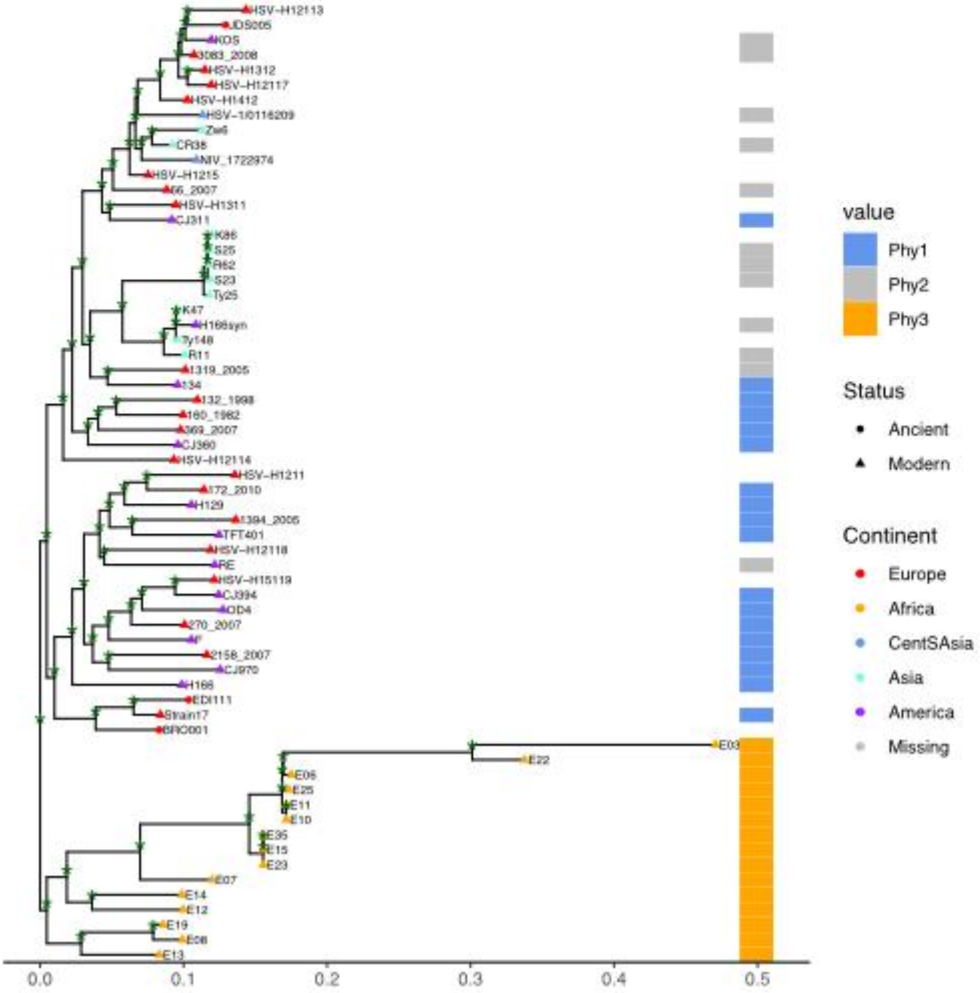

Fig. S3. Maximum Likelihood Phylogeny. Maximum likelihood phylogenetic tree over the HSV-1 dataset. Tip colours provide the continent of sampling, with the three ancient samples shown with circles and all other tips (modern samples) having triangular symbols. Panel at right provides the phylogroup assignment for those samples overlapping with Pfaff *et al.* 2016 as denoted in the legend. * denote nodes with >70% boot-strap support following 1000 boot-strap iterations of the tree building step.

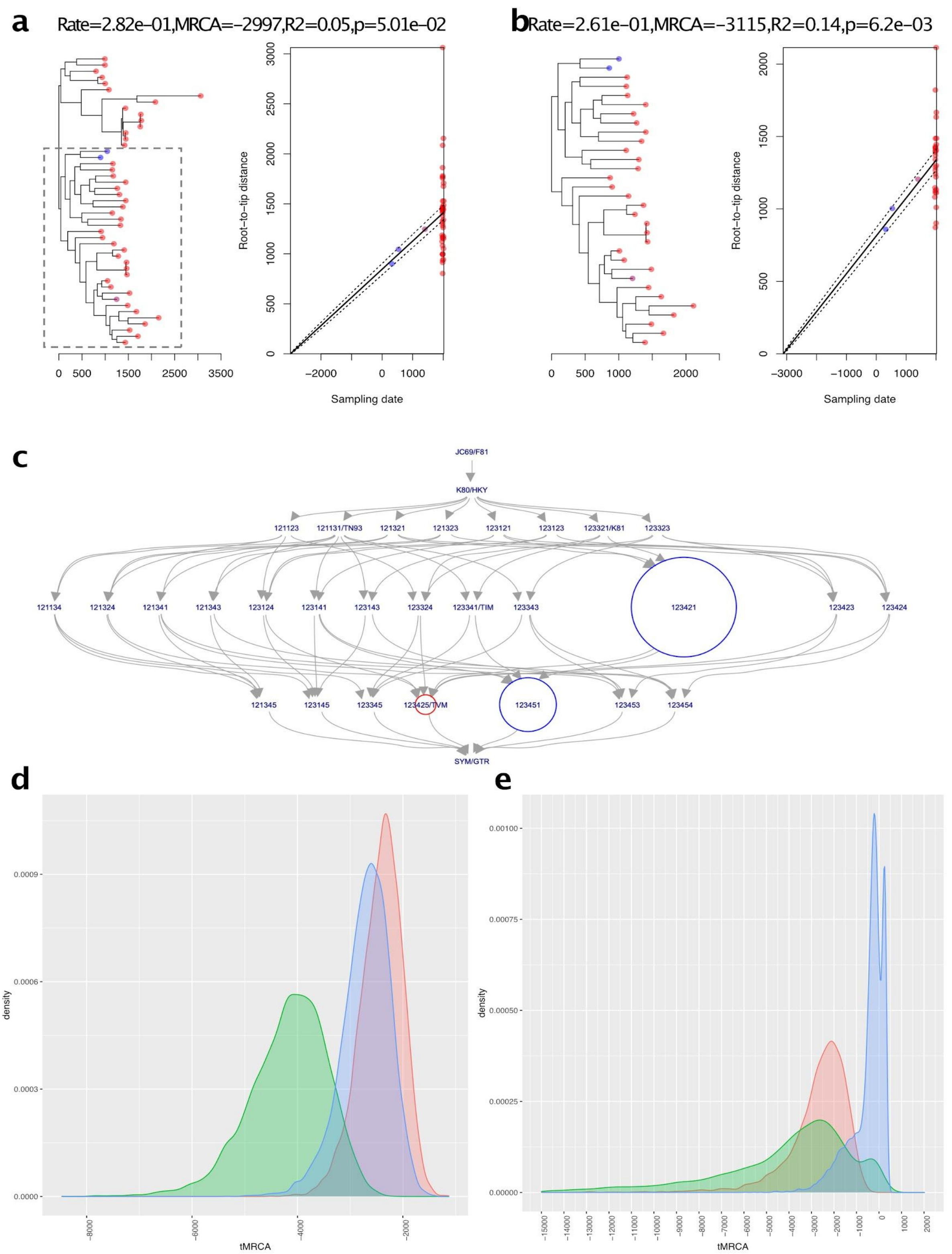

Fig. S4. Testing for temporal signal, BModelTest and Posterior Densities. (**A**), Linear relationship between the time of sample collection (x-axis) and root-to-tip phylogenetic distance (y-axis) estimated from a recombination pruned maximum likelihood phylogenetic tree over the global phylogenetic dataset. Confidence intervals are indicated by dashed lines. Colouration of points denotes age of sample from oldest (blue) to more recent (red). (**B**),

Following identification of a significant temporal signal at the node falling basal to the non-African HSV-1, the same plot is demonstrated only for descendants from this node (as highlighted in panel a. In both cases plot headings provide the rate, MRCA, regression coefficient and p-value following 10,000 permutations of the sampling date computed using the BactDating roottotip() function. (**C**), Models with blue circles are inside 95% HPD, red outside, and without circles have at most 0.40% support. Model 123421 (transversion model – TVM) had 86.79% posterior support and 86.79% cumulative support. (**D**), Posterior distributions estimated under a strict clock and three possible specifications of demographic models (red - coalescent constant, green - coalescent exponential, blue - coalescent skyline). Estimated tree heights are given in the left-hand panels, estimated clock rates are provided in the right-hand panels. (**E**), Posterior distributions estimated under a relaxed clock and three possible specifications of demographic models (red - coalescent constant, green - coalescent exponential, blue - coalescent skyline). Estimated tree heights are given in the left-hand panels, estimated clock rates are provided in the right-hand panels.

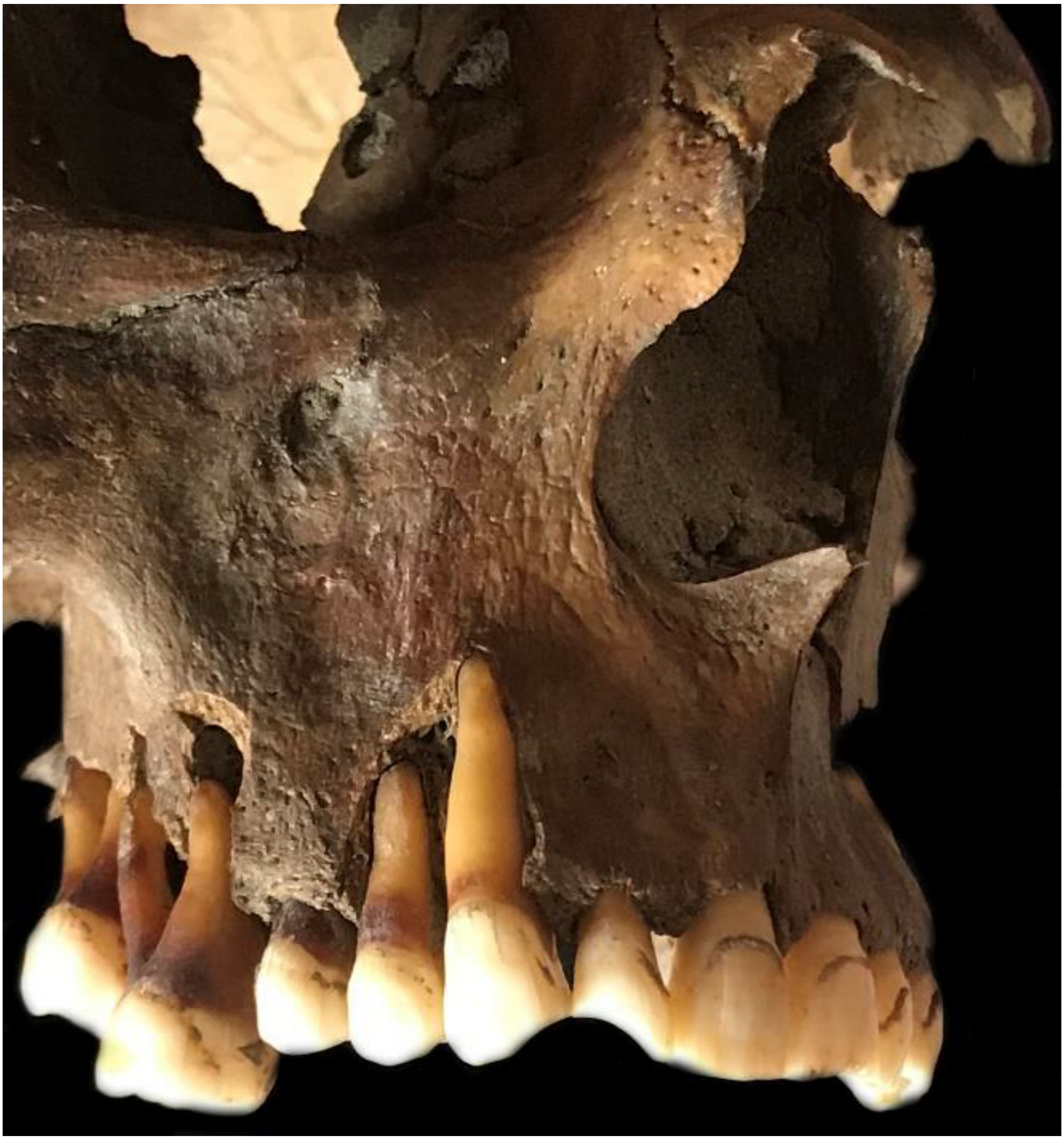

Fig. S5. Individual S16, male, 26-35 years old. The man was a fervent smoker of clay pipes. Traces of the habit are visible in multiple places on the teeth, where the hard clay pipe, usually put in the same place in the mouth, has worn the teeth.

Table S1. Summary of contamination estimates from human genomes. Summary of contamination estimates utilised in this study including mtDNA-based, and X-based for males over 1x coverage.

ID	mtDNA based contam.	X chrom. based contam.	SNP sites	SNP sites with flanking	Method1: old		Method1: new		Method2: old		Method2: new	
					MoM	ML	MoM	ML	MoM	ML	MoM	ML
JDS005	0.21%	0.69%	54,748	492,732	0.7763% ± 4.8e-4	0.6023% ± 6.5e-14	0.7705% ± 4.8e-4	0.6027% ± 7.0e-13	0.7721% ± 1.1e-3	0.6284% ± 1.3e-13	0.7663% ± 1.1e-3	0.6280% ± 1.7e-12
EDI111	0.17%	N/A										
BRO001	0.06%	N/A										
RIJ001	1.05%	N/A										

X-based contamination estimates can only be run on males with sufficient autosomal coverage, in this case only JDS005 fit the criteria.

Table S2. Runs of homozygosity in samples over 1x genomic coverage. Summary of output from HapROH (*115*).

Sample ID	max_roh (cM)	sum_roh (>0.1cM)	n_roh (>0.1cM)	sum_roh (>0.5cM)	n_roh (>0.5cM)	sum_roh (>1cM)	n_roh (>1cM)	sum_roh (>2cM)	n_roh (>2cM)	sum_roh (>4cM)	n_roh (>4cM)	sum_roh (>8cM)	roh (>8c
JDS005	2.15	70.68	55	70.68	55	70.68	55	4.21	2	0.00	0	0.00	0
EDI111	2.82	77.16	59	77.16	59	77.16	59	4.87	2	0.00	0	0.00	0

Note: max_roh: the longest ROH segment (cM), n_roh: total number of ROH segments, sum_roh: total length of ROH segments.

Table S3. BEAST2 posterior estimates following assessment of the results over three demographic models and tested using both strict and relaxed priors. Posterior, likelihood, tree height, clock rate, marginal likelihood and standard deviation (SD) for three models under strict and relaxed clock priors.

Strict Clock						
Demographic model	Posterior_ESS	Likelihood	TreeHeight	ClockRate	Marginal_Likelihood	SD
Coalescent_constant	24303.305	-191833.726(-191843.326--191825.36)	6138.328(4844.956-7830.772)	7.400e-07(5.568e-07-9.160e-07)	-192077.699	18.39
Coalescent_exponential	2109.163	-191834.328(-191843.986--191825.872)	8063.489(5982.904-10795.248)	5.542e-07(3.930e-07-7.179e-07)	-192127.163	18.20
Coalescent_Bayes_Sky	22405.288	-191795.129(-191804.854--191786.913)	7268.874(5477.789-9591.929)	6.052e-07(4.380e-07-7.773e-07)	-192048.638	16.93

Relaxed Clock						
Demographic model	Posterior_ESS	Likelihood	TreeHeight	ClockRate	Marginal_Likelihood	SD
Coalescent_constant	2507.823	-191627.308(-191639.602--191616.991)	4634.172(2524.156-9330.682)	1.028e-06(3.611e-07-1.752e-06)	-193976.069	45
Coalescent_exponential	*82.523*	*-191788.646(-191800.533--191777.469)*	*9903.537(3846.677-22354.934)*	*4.560e-07(1.4589e-07-9.361e-07)*	*/*	*/*
Coalescent_Bayes_Sky	3243.329	-191642.994(-191654.969--191632.129)	13834.335(3992.156-102241.329)	3.3691e-07(4.373e-11-7.322e-07)	-193603.489	41

Note: Marginal likelihood and SD estimates are also provided. The analysis highlighted in italics failed to converge following 200 million iterations.

Table S4. Results of genotyping of HSV-1 strains included in this study.

Gene/Publication	SNP	JDS005		EDI111		BRO001		RIJ001	
		Base	Group	Base	Group	Base	Group	Base	Group
US2 (Glück et al. 2015)	134171-5	GGGAT	A/B	GGGAT	A/B	GGGAC	C	*N/A*	-
	134185	A/G*	A/B/C	A	A	G	A/B/C	*N/A*	-
	134217	*N/A*	-	A	A	G	B/C	G	B/C
	134237	??G*	A	*N/A*	-	CCT	B/C	CCT	B/C
	134241	A*	A	*N/A*	-	G	B/C	G	B/C
US4/gG (Norberg et al. 2006)	137088	*N/A*	-	*N/A*	-	*N/A*	-	*T*	B/C
	137090	*N/A*	-	*N/A*	-	*N/A*	-	*A*	B/C
	136974	C*	A/B	T*	C	C		C	A/B
US7/gI (Norberg et al. 2006)	140160	G	A/B	G	A/B	*N/A*	-	*G*	A/B
	140298	T	A	G*	B/C	G	B/C	G*	B/C
Result			A/(A,B)?/A		A/C/B		C/?/(B,C?)		(B,C)/B/B
* Only 1X coverage at this position									
N/A: No coverage at the position of interest.									

Data S1. (separate file)

Sequencing and mapping statistics for human and HSV-1 genomes alignments.
A - List of sequencing technology, kits and raw output statistics and mapping statistics for human and B - HSV-1 genome alignments.

Data S2. (separate file)

Herpes infection-related host susceptibility variants
List of variants within genes of interest regarding Herpes susceptibility and presence in the host genomes.

Data S3. (separate file)

Host genotypes annotated with ClinVar database.
List of variants present in JDS005 and EDI111 annotated by ClinVar. Includes directly called and imputed genotypes.

Data S4. (separate file)

Metagenomic profiles of periodontal disease-related bacteria.
KrakenUniq summary statistics for periodontal disease-related species targeted in this study.

Data S5. (separate file)

Results of SNP effect analysis for ancient HSV-1 strains
Results of the snpEff analysis. Each tab is the output for an ancient strain.

Data S6. (separate file)
Gene interval coverage and mappability estimates
Summary of interval coverage and mappability for genes in each HSV-1 genome generated in this study.

Data S7. (separate file)

List of comparative HSV genomes
Details for comparative HSV-1 genomes assessed for this study.

Data S8. (separate file)

Recombination breakpoints and associated significance
List of recombination breakpoints and associated significance assessed following 3Seq analysis applied to the core genome alignment.

Data S9. (separate file)

Radiocarbon dating information and archaeological dates.
Yield and calibration results for newly generated radiocarbon dates and relative chronology dating.

.

REFERENCES AND NOTES

1. B. Mühlemann, T. C. Jones, P. de Barros Damgaard, M. E. Allentoft, I. Shevnina, A. Logvin, E. Usmanova, I. P. Panyushkina, B. Boldgiv, T. Bazartseren, K. Tashbaeva, V. Merz, N. Lau, V. Smrčka, D. Voyakin, E. Kitov, A. Epimakhov, D. Pokutta, M. Vicze, T. D. Price, V. Moiseyev, A. J. Hansen, L. Orlando, S. Rasmussen, M. Sikora, L. Vinner, A. D. M. E. Osterhaus, D. J. Smith, D. Glebe, R. A. M. Fouchier, C. Drosten, K.-G. Sjögren, K. Kristiansen, E. Willerslev, Ancient hepatitis B viruses from the Bronze Age to the Medieval period. *Nature* **557**, 418–423 (2018).

2. K. I. Bos, K. M. Harkins, A. Herbig, M. Coscolla, N. Weber, I. Comas, S. A. Forrest, J. M. Bryant, S. R. Harris, V. J. Schuenemann, T. J. Campbell, K. Majander, A. K. Wilbur, R. A. Guichon, D. L. Wolfe Steadman, D. C. Cook, S. Niemann, M. A. Behr, M. Zumarraga, R. Bastida, D. Huson, K. Nieselt, D. Young, J. Parkhill, J. E. Buikstra, S. Gagneux, A. C. Stone, J. Krause, Pre-Columbian mycobacterial genomes reveal seals as a source of New World human tuberculosis. *Nature* **514**, 494–497 (2014).

3. Herpes simplex virus; www.who.int/news-room/fact-sheets/detail/herpes-simplex-virus.

4. R. Whitley, D. W. Kimberlin, C. G. Prober, in *Human Herpesviruses: Biology, Therapy, and Immunoprophylaxis*, A. Arvin, G. Campadelli-Fiume, E. Mocarski, P. S. Moore, B. Roizman, R. Whitley, K. Yamanishi, Eds. (Cambridge Univ. Press, 2011).

5. C. H. Andrewes, E. Arnold Carmichael, A note on the presence of antibodies to herpes virus: In post-encephalitic and other human sera. *Lancet* **215**, 857–858 (1930).

6. J. R. Baringer, P. Swoveland, Recovery of herpes-simplex virus from human trigeminal ganglions. *N. Engl. J. Med.* **288**, 648–650 (1973).

7. E. Criscuolo, M. Castelli, R. A. Diotti, V. Amato, R. Burioni, M. Clementi, A. Ambrosi, N. Mancini, N. Clementi, Cell-to-cell spread blocking activity is extremely limited in the sera of herpes simplex virus 1 (HSV-1)- and HSV-2-infected subjects. *J. Virol.* **93**, e00070-19 (2019).

8. M. Moraru, E. Cisneros, N. Gómez-Lozano, R. de Pablo, F. Portero, M. Cañizares, M. Vaquero, G. Roustán, I. Millán, M. López-Botet, C. Vilches, Host genetic factors in susceptibility to herpes simplex

type 1 virus infection: Contribution of polymorphic genes at the interface of innate and adaptive immunity. *J. Immunol.* **188**, 4412–4420 (2012).

9. S.-Y. Zhang, E. Jouanguy, S. Ugolini, A. Smahi, G. Elain, P. Romero, D. Segal, V. Sancho-Shimizu, L. Lorenzo, A. Puel, C. Picard, A. Chapgier, S. Plancoulaine, M. Titeux, C. Cognet, H. von Bernuth, C.-L. Ku, A. Casrouge, X.-X. Zhang, L. Barreiro, J. Leonard, C. Hamilton, P. Lebon, B. Héron, L. Vallée, L. Quintana-Murci, A. Hovnanian, F. Rozenberg, E. Vivier, F. Geissmann, M. Tardieu, L. Abel, J.-L. Casanova, TLR3 deficiency in patients with herpes simplex encephalitis. *Science* **317**, 1522–1527 (2007).

10. C. J. Houldcroft, Human herpesvirus sequencing in the genomic era: The growing ranks of the herpetic legion. *Pathogens* **8**, 186 (2019).

11. F. Pfaff, M. Groth, A. Sauerbrei, R. Zell, Genotyping of herpes simplex virus type 1 by whole-genome sequencing. *J. Gen. Virol.* **97**, 2732–2741 (2016).

12. C. D. Bowen, H. Paavilainen, D. W. Renner, J. Palomäki, J. Lehtinen, T. Vuorinen, P. Norberg, V. Hukkanen, M. L. Szpara, Comparison of herpes simplex virus 1 strains circulating in Finland demonstrates the uncoupling of whole-genome relatedness and phenotypic outcomes of viral infection. *J. Virol.* **93**, e01824-18 (2019).

13. M. L. Szpara, D. Gatherer, A. Ochoa, B. Greenbaum, A. Dolan, R. J. Bowden, L. W. Enquist, M. Legendre, A. J. Davison, Evolution and diversity in human herpes simplex virus genomes. *J. Virol.* **88**, 1209–1227 (2014).

14. A. W. Kolb, C. Ané, C. R. Brandt, Using HSV-1 genome phylogenetics to track past human migrations. *PLOS ONE* **8**, e76267 (2013).

15. H. U. Bernard, Coevolution of papiliomaviruses with human populations. *Trends Microbiol.* **2**, 140–143 (1994).

16. I. I. Podgorski, L. Pantó, K. Földes, I. de Winter, M. Jánoska, E. Sós, B. Chenet, B. Harrach, M. Benkő, Adenoviruses of the most ancient primate lineages support the theory on virus-host co-evolution. *Acta Vet. Hung.* **66**, 474–487 (2018).

17. S. H. Nielsen, L. van Dorp, C. J. Houldcroft, A. G. Pedersen, M. E. Allentoft, L. Vinner, A. Margaryan, E. Pavlova, V. Chasnyk, P. Nikolskiy, V. Pitulko, V. N. Pimenoff, F. Balloux, M. Sikora, 31,600-year-old human virus genomes support a Pleistocene origin for common childhood infections. *bioRxiv* 2021.06.28.450199 (2021).

18. D. Forni, C. Pontremoli, M. Clerici, U. Pozzoli, R. Cagliani, M. Sironi, Recent out-of-Africa migration of human herpes simplex viruses. *Mol. Biol. Evol.* **37**, 1259–1271 (2020).

19. A. L. Greninger, P. Roychoudhury, H. Xie, A. Casto, A. Cent, G. Pepper, D. M. Koelle, M.-L. Huang, A. Wald, C. Johnston, K. R. Jerome, Ultrasensitive capture of human herpes simplex virus genomes directly from clinical samples reveals extraordinarily limited evolution in cell culture. *mSphere* **3**, e00283–18 (2018).

20. M. M. Shipley, D. W. Renner, M. Ott, D. C. Bloom, D. M. Koelle, C. Johnston, M. L. Szpara, Genome-wide surveillance of genital herpes simplex virus type 1 from multiple anatomic sites over time. *J. Infect. Dis.* **218**, 595–605 (2018).

21. K. Lee, A. W. Kolb, Y. Sverchkov, J. A. Cuellar, M. Craven, C. R. Brandt, Recombination analysis of herpes simplex virus 1 reveals a bias toward GC content and the inverted repeat regions. *J. Virol.* **89**, 7214–7223 (2015).

22. S. Flexner, H. L. Amoss, Contributions to the pathology of experimental virus encephalitis: III. Varieties and properties of the herpes virus. *J. Exp. Med.* **41**, 357–377 (1925).

23. E. C. Holmes, Evolutionary history and phylogeography of human viruses. *Annu. Rev. Microbiol.* **62**, 307–328 (2008).

24. P. Aiewsakun, A. Katzourakis, Time-dependent rate phenomenon in viruses. *J. Virol.* **90**, 7184–7195 (2016).

25. M. A. Spyrou, K. I. Bos, A. Herbig, J. Krause, Ancient pathogen genomics as an emerging tool for infectious disease research. *Nat. Rev. Genet.* **20**, 323–340 (2019).

26. D. Juhl, C. Mosel, F. Nawroth, A.-M. Funke, S. M. Dadgar, H. Hagenström, H. Kirchner, H. Hennig, Detection of herpes simplex virus DNA in plasma of patients with primary but not with recurrent infection: Implications for transfusion medicine? *Transfus. Med.* **20**, 38–47 (2010).

27. S. Zhong, A. Naqvi, E. Bair, S. Nares, A. A. Khan, Viral microRNAs identified in human dental pulp. *J. Endod.* **43**, 84–89 (2017).

28. M. M. A. G. Kazi, R. Bharadwaj, K. Bhat, D. Happy, Association of herpes viruses with mild, moderate and severe chronic periodontitis. *J. Clin. Diagn. Res.* **9**, DC05–DC08 (2015).

29. A. Szolek, B. Schubert, C. Mohr, M. Sturm, M. Feldhahn, O. Kohlbacher, OptiType: Precision HLA typing from next-generation sequencing data. *Bioinformatics* **30**, 3310–3316 (2014).

30. A. A. Alzahrani, Association between human herpes virus and aggressive periodontitis: A systematic review. *Saudi J. Dent. Res.* **8**, 97–104 (2017).

31. H. Silva, Tobacco use and periodontal disease–The role of microvascular dysfunction. *Biology* **10**, 441 (2021).

32. S. S. Socransky, A. D. Haffajee, M. A. Cugini, C. Smith, R. L. Kent Jr., Microbial complexes in subgingival plaque. *J. Clin. Periodontol.* **25**, 134–144 (1998).

33. A. H. Walton, J. T. Muenzer, D. Rasche, J. S. Boomer, B. Sato, B. H. Brownstein, A. Pachot, T. L. Brooks, E. Deych, W. D. Shannon, J. M. Green, G. A. Storch, R. S. Hotchkiss, Reactivation of multiple viruses in patients with sepsis. *PLOS ONE* **9**, e98819 (2014).

34. A. J. Rutkowski, F. Erhard, A. L'Hernault, T. Bonfert, M. Schilhabel, C. Crump, P. Rosenstiel, S. Efstathiou, R. Zimmer, C. C. Friedel, L. Dölken, Widespread disruption of host transcription termination in HSV-1 infection. *Nat. Commun.* **6**, 7126 (2015).

35. D. H. Huson, D. Bryant, Application of phylogenetic networks in evolutionary studies. *Mol. Biol. Evol.* **23**, 254–267 (2006).

36. D. H. Alexander, J. Novembre, K. Lange, Fast model-based estimation of ancestry in unrelated individuals. *Genome Res.* **19**, 1655–1664 (2009).

37. D. J. Lawson, G. Hellenthal, S. Myers, D. Falush, Inference of population structure using dense haplotype data. *PLOS Genet.* **8**, e1002453 (2012).

38. D. M. Koelle, P. Norberg, M. P. Fitzgibbon, R. M. Russell, A. L. Greninger, M.-L. Huang, L. Stensland, L. Jing, A. S. Magaret, K. Diem, S. Selke, H. Xie, C. Celum, J. R. Lingappa, K. R. Jerome, A. Wald, C. Johnston, Worldwide circulation of HSV-2 × HSV-1 recombinant strains. *Sci. Rep.* **7**, 44084 (2017).

39. S. Burrel, D. Boutolleau, D. Ryu, H. Agut, K. Merkel, F. H. Leendertz, S. Calvignac-Spencer, Ancient recombination events between human herpes simplex viruses. *Mol. Biol. Evol.* **34**, 1713–1721 (2017).

40. D. P. Martin, A. Varsani, P. Roumagnac, G. Botha, S. Maslamoney, T. Schwab, Z. Kelz, V. Kumar, B. Murrell, RDP5: A computer program for analyzing recombination in, and removing signals of recombination from, nucleotide sequence datasets. *Virus Evol.* **7**, veaa087 (2021).

41. R. Bouckaert, T. G. Vaughan, J. Barido-Sottani, S. Duchêne, M. Fourment, A. Gavryushkina, J. Heled, G. Jones, D. Kühnert, N. De Maio, M. Matschiner, F. K. Mendes, N. F. Müller, H. A. Ogilvie, L. du Plessis, A. Popinga, A. Rambaut, D. Rasmussen, I. Siveroni, M. A. Suchard, C.-H. Wu, D. Xie, C. Zhang, T. Stadler, A. J. Drummond, BEAST 2.5: An advanced software platform for Bayesian evolutionary analysis. *PLOS Comput. Biol.* **15**, e1006650 (2019).

42. E. Hedner, A. Vahlne, K. E. Kahnberg, J. M. Hirsch, Reactivated herpes simplex virus infection as a possible cause of dry socket after tooth extraction. *J. Oral Maxillofac. Surg.* **51**, 370–376 (1993).

43. K. Held, T. Derfuss, Control of HSV-1 latency in human trigeminal ganglia—Current overview. *J. Neurovirol.* **17**, 518–527 (2011).

44. M. E. Allentoft, M. Sikora, K.-G. Sjögren, S. Rasmussen, M. Rasmussen, J. Stenderup, P. B. Damgaard, H. Schroeder, T. Ahlström, L. Vinner, A.-S. Malaspinas, A. Margaryan, T. Higham, D. Chivall, N. Lynnerup, L. Harvig, J. Baron, P. Della Casa, P. Dąbrowski, P. R. Duffy, A. V. Ebel, A. Epimakhov, K. Frei, M. Furmanek, T. Gralak, A. Gromov, S. Gronkiewicz, G. Grupe, T. Hajdu, R.

Jarysz, V. Khartanovich, A. Khokhlov, V. Kiss, J. Kolář, A. Kriiska, I. Lasak, C. Longhi, G. McGlynn, A. Merkevicius, I. Merkyte, M. Metspalu, R. Mkrtchyan, V. Moiseyev, L. Paja, G. Pálfi, D. Pokutta, Ł. Pospieszny, T. D. Price, L. Saag, M. Sablin, N. Shishlina, V. Smrčka, V. I. Soenov, V. Szeverényi, G. Tóth, S. V. Trifanova, L. Varul, M. Vicze, L. Yepiskoposyan, V. Zhitenev, L. Orlando, T. Sicheritz-Pontén, S. Brunak, R. Nielsen, K. Kristiansen, E. Willerslev, Population genomics of Bronze Age Eurasia. *Nature* **522**, 167–172 (2015).

45. I. Feeser, W. Dörfler, J. Kneisel, M. Hinz, S. Dreibrodt, Human impact and population dynamics in the Neolithic and Bronze Age: Multi-proxy evidence from north-western Central Europe. *Holocene* **29**, 1596–1606 (2019).

46. L. A. Weinert, D. P. Depledge, S. Kundu, A. A. Gershon, R. A. Nichols, F. Balloux, J. J. Welch, J. Breuer, Rates of vaccine evolution show strong effects of latency: Implications for varicella zoster virus epidemiology. *Mol. Biol. Evol.* **32**, 1020–1028 (2015).

47. I. Comas, M. Coscolla, T. Luo, S. Borrell, K. E. Holt, M. Kato-Maeda, J. Parkhill, B. Malla, S. Berg, G. Thwaites, D. Yeboah-Manu, G. Bothamley, J. Mei, L. Wei, S. Bentley, S. R. Harris, S. Niemann, R. Diel, A. Aseffa, Q. Gao, D. Young, S. Gagneux, Out-of-Africa migration and Neolithic coexpansion of *Mycobacterium tuberculosis* with modern humans. *Nat. Genet.* **45**, 1176–1182 (2013).

48. F. Menardo, S. Duchêne, D. Brites, S. Gagneux, The molecular clock of *Mycobacterium tuberculosis*. *PLOS Pathog.* **15**, e1008067 (2019).

49. C. D. Bowen, D. W. Renner, J. T. Shreve, Y. Tafuri, K. M. Payne, R. D. Dix, P. R. Kinchington, D. Gatherer, M. L. Szpara, Viral forensic genomics reveals the relatedness of classic herpes simplex virus strains KOS, KOS63, and KOS79. *Virology* **492**, 179–186 (2016).

50. C. L. Scheib, H. Li, T. Desai, V. Link, C. Kendall, G. Dewar, P. W. Griffith, A. Mörseburg, J. R. Johnson, A. Potter, S. L. Kerr, P. Endicott, J. Lindo, M. Haber, Y. Xue, C. Tyler-Smith, M. S. Sandhu, J. G. Lorenz, T. D. Randall, Z. Faltyskova, L. Pagani, P. Danecek, T. C. O'Connell, P. Martz, A. S. Boraas, B. F. Byrd, A. Leventhal, R. Cambra, R. Williamson, L. Lesage, B. Holguin, E. Ygnacio-De Soto, J. Rosas, M. Metspalu, J. T. Stock, A. Manica, A. Scally, D. Wegmann, R. S. Malhi, T. Kivisild,

Ancient human parallel lineages within North America contributed to a coastal expansion. *Science* **360**, 1024–1027 (2018).

51. M. Guellil, M. Keller, J. M. Dittmar, S. A. Inskip, C. Cessford, A. Solnik, T. Kivisild, M. Metspalu, J. E. Robb, C. L. Scheib, An invasive *Haemophilus influenzae* serotype B infection in an Anglo-Saxon plague victim. *Genome Biol.* **23**, 22 (2022).

52. L. Saag, M. Laneman, L. Varul, M. Malve, H. Valk, M. A. Razzak, I. G. Shirobokov, V. I. Khartanovich, E. R. Mikhaylova, A. Kushniarevich, C. L. Scheib, A. Solnik, T. Reisberg, J. Parik, L. Saag, E. Metspalu, S. Rootsi, F. Montinaro, M. Remm, R. Mägi, E. D'Atanasio, E. R. Crema, D. Díez-Del-Molino, M. G. Thomas, A. Kriiska, T. Kivisild, R. Villems, V. Lang, M. Metspalu, K. Tambets, The arrival of siberian ancestry connecting the eastern Baltic to Uralic speakers further East. *Curr. Biol.* **29**, 1701–1711.e16 (2019).

53. D. E. Wood, S. L. Salzberg, Kraken: Ultrafast metagenomic sequence classification using exact alignments. *Genome Biol.* **15**, R46 (2014).

54. H. Li, B. Handsaker, A. Wysoker, T. Fennell, J. Ruan, N. Homer, G. Marth, G. Abecasis, R. Durbin; 1000 genome project data processing subgroup, The sequence alignment/map format and SAMtools. *Bioinformatics* **25**, 2078–2079 (2009).

55. H. Jónsson, A. Ginolhac, M. Schubert, P. L. F. Johnson, L. Orlando, mapDamage2.0: Fast approximate Bayesian estimates of ancient DNA damage parameters. *Bioinformatics* **29**, 1682–1684 (2013).

56. F. P. Breitwieser, D. N. Baker, S. L. Salzberg, KrakenUniq: Confident and fast metagenomics classification using unique *k*-mer counts. *Genome Biol.* **19**, 198 (2018).

57. H. Li, R. Durbin, Fast and accurate long-read alignment with Burrows-Wheeler transform. *Bioinformatics* **26**, 589–595 (2010).

58. Picard Tools - By Broad Institute; http://broadinstitute.github.io/picard/.

59. G. A. Van der Auwera, M. O. Carneiro, C. Hartl, R. Poplin, G. Del Angel, A. Levy-Moonshine, T. Jordan, K. Shakir, D. Roazen, J. Thibault, E. Banks, K. V. Garimella, D. Altshuler, S. Gabriel, M. A.

DePristo, From FastQ data to high confidence variant calls: The Genome Analysis Toolkit best practices pipeline. *Curr. Protoc. Bioinformatics* **43**, 11.10.1–11.10.33 (2013).

60. M. Guellil, *MeriamGuellil/aDNA-BAMPlotter: ADNA BAM plotter script for microbial genomes* (2021); https,//zenodo.org/record/5676093.

61. K. Okonechnikov, A. Conesa, F. García-Alcalde, Qualimap 2: Advanced multi-sample quality control for high-throughput sequencing data. *Bioinformatics* **32**, 292–294 (2016).

62. C. Pockrandt, M. Alzamel, C. S. Iliopoulos, K. Reinert, GenMap: Fast and exact computation of genome mappability. *bioRxiv* 611160 (2019).

63. A. R. Quinlan, I. M. Hall, BEDTools: A flexible suite of utilities for comparing genomic features. *Bioinformatics* **26**, 841–842 (2010).

64. P. Cingolani, A. Platts, L. L. Wang, M. Coon, T. Nguyen, L. Wang, S. J. Land, X. Lu, D. M. Ruden, A program for annotating and predicting the effects of single nucleotide polymorphisms, SnpEff: SNPs in the genome of *Drosophila melanogaster* strain w1118; iso-2; iso-3. *Fly* **6**, 80–92 (2012).

65. P. Norberg, T. Bergström, J.-A. Liljeqvist, Genotyping of clinical herpes simplex virus type 1 isolates by use of restriction enzymes. *J. Clin. Microbiol.* **44**, 4511–4514 (2006).

66. B. Glück, S. Möbius, F. Pfaff, R. Zell, A. Sauerbrei, Novel method for genotyping clinical herpes simplex virus type 1 isolates. *Arch. Virol.* **160**, 2807–2811 (2015).

67. D. Alexander, K. Lange, Enhancements to the ADMIXTURE algorithm for individual ancestry estimation. *BMC Bioinformatics* **12**, 246 (2011).

68. R. T. Sarisky, T. T. Nguyen, K. E. Duffy, R. J. Wittrock, J. J. Leary, Difference in incidence of spontaneous mutations between herpes simplex virus types 1 and 2. *Antimicrob. Agents Chemother.* **44**, 1524–1529 (2000).

69. F. Lassalle, M. A. Beale, T. Bharucha, C. A. Williams, R. J. Williams, J. Cudini, R. Goldstein, T. Haque, D. P. Depledge, J. Breuer, Whole genome sequencing of herpes simplex virus 1 directly from

human cerebrospinal fluid reveals selective constraints in neurotropic viruses. *Virus Evol.* **6**, veaa012 (2020).

70. L.-T. Nguyen, H. A. Schmidt, A. von Haeseler, B. Q. Minh, IQ-TREE: A fast and effective stochastic algorithm for estimating maximum-likelihood phylogenies. *Mol. Biol. Evol.* **32**, 268–274 (2015).

71. N. J. Croucher, A. J. Page, T. R. Connor, A. J. Delaney, J. A. Keane, S. D. Bentley, J. Parkhill, S. R. Harris, Rapid phylogenetic analysis of large samples of recombinant bacterial whole genome sequences using Gubbins. *Nucleic Acids Res.* **43**, e15 (2015).

72. G. Yu, D. K. Smith, H. Zhu, Y. Guan, T. T. Lam, GGTREE: An R package for visualization and annotation of phylogenetic trees with their covariates and other associated data. *Methods Ecol. Evol.* **8**, 28–36 (2017).

73. R. C. Edgar, MUSCLE: Multiple sequence alignment with high accuracy and high throughput. *Nucleic Acids Res.* **32**, 1792–1797 (2004).

74. A. Doizy, A. Prin, G. Cornu, F. Chiroleu, A. Rieux, PhyloSTemS: A new graphical tool to investigate temporal signal of heterochronous sequences at various evolutionary scales. *Authorea Preprints*, 10.22541/au.159050344.45788558 (2020).

75. X. Didelot, N. J. Croucher, S. D. Bentley, S. R. Harris, D. J. Wilson, Bayesian inference of ancestral dates on bacterial phylogenetic trees. *Nucleic Acids Res.* **46**, e134 (2018).

76. R. R. Bouckaert, A. J. Drummond, bModelTest: Bayesian phylogenetic site model averaging and model comparison. *BMC Evol. Biol.* **17**, 42 (2017).

77. P. M. Russel, B. J. Brewer, S. Klaere, R. R. Bouckaert, Model selection and parameter inference in phylogenetics using nested sampling. *Syst. Biol.* **68**, 219–233 (2019).

78. T. S. Korneliussen, A. Albrechtsen, R. Nielsen, ANGSD: Analysis of next generation sequencing data. *BMC Bioinformatics* **15**, 356 (2014).

79. R. Hui, E. D'Atanasio, L. M. Cassidy, C. L. Scheib, T. Kivisild, Evaluating genotype imputation pipeline for ultra-low coverage ancient genomes. *Sci. Rep.* **10**, 18542 (2020).

80. C. Firth, A. Kitchen, B. Shapiro, M. A. Suchard, E. C. Holmes, A. Rambaut, Using time-structured data to estimate evolutionary rates of double-stranded DNA viruses. *Mol. Biol. Evol.* **27**, 2038–2051 (2010).

81. M. Rubin, *Charity and Community in Medieval Cambridge* (Cambridge Univ. Press, 1987).

82. C. Cessford, The St. John's Hospital cemetery and environs, Cambridge: Contextualizing the Medieval urban dead. *Archaeol. J.* **172**, 52–120 (2015).

83. T. Malim, J. Hines, *The Anglo Saxon Cemetery at Edix Hill Barrington A, Cambridgeshire* (CBA report 112; Council for British Archaeology, York, 1998).

84. V. F. Gening, Бродовский могильник. *КСИИМК*, 87 (1953).

85. R. D. Goldina, Голдина РД О датировке и хронологии неволинской культуры (конец IV--начало IX в. н. э.). *Древности Прикамья эпохи железа (VI в. до н. э. --XV в. н. э.): хронологическая атрибуция. Ed. RD Goldina. Удмуртский университет, Ижевск*, 203–285 (2012).

86. A. M. H. C. Koekkelkoren, B. Veselka, M. C. van Dam, A. A. J. Griffioen, M. Vitters, A. W. E. Wilbers, D. F. A. M. van den Biggelaar, E. Hees, Y. van Amerongen, L. Kootker, S. W. L. Palstra, Opgraving variant Archeologische Begeleiding Raadhuisstraat 185-193, Alphen aan den Rijn, Gemeente Alphen aan den Rijn.IDDS Archeologie Rapport, No. 2313 (2021).

87. E. Goldina, R. Goldina, On North-Western contacts of perm Finns in VII–VIII centuries. *Estonian J. Archaeol.* **22**, 163 (2018).

88. A. J. Forrester, V. Sullivan, A. Simmons, B. A. Blacklaws, G. L. Smith, A. A. Nash, A. C. Minson, Induction of protective immunity with antibody to herpes simplex virus type 1 glycoprotein H (gH) and analysis of the immune response to gH expressed in recombinant vaccinia virus. *J. Gen. Virol.* **72**, 369–375 (1991).

89. C. Parry, S. Bell, T. Minson, H. Browne, Herpes simplex virus type 1 glycoprotein H binds to αvβ3 integrins. *J. Gen. Virol.* **86**, 7–10 (2005).

90. J. C. Whitbeck, Z.-Y. Huang, T. M. Cairns, J. R. Gallagher, H. Lou, M. Ponce-de-Leon, R. B. Belshe, R. J. Eisenberg, G. H. Cohen, Repertoire of epitopes recognized by serum IgG from humans vaccinated with herpes simplex virus 2 glycoprotein D. *J. Virol.* **88**, 7786–7795 (2014).

91. P. Danecek, A. Auton, G. Abecasis, C. A. Albers, E. Banks, M. A. DePristo, R. E. Handsaker, G. Lunter, G. T. Marth, S. T. Sherry, G. McVean, R. Durbin; 1000 Genomes Project Analysis Group, The variant call format and VCFtools. *Bioinformatics* **27**, 2156–2158 (2011).

92. M. M. A. G. Kazi, R. Bharadwaj, Role of herpesviruses in chronic periodontitis and their association with clinical parameters and in increasing severity of the disease. *Eur. J. Dent.* **11**, 299–304 (2017).

93. C. Zhu, F. Li, M. C. M. Wong, X.-P. Feng, H.-X. Lu, W. Xu, Association between herpesviruses and chronic periodontitis: A meta-analysis based on case-control studies. *PLOS ONE* **10**, e0144319 (2015).

94. R. E. M. Hedges, L. M. Reynard, Nitrogen isotopes and the trophic level of humans in archaeology. *J. Archaeol. Sci.* **34**, 1240–1251 (2007).

95. J. A. Lee-Thorp, On isotopes and old bones. *Archaeometry* **50**, 925–950 (2008).

96. M. J. DeNiro, S. Epstein, Influence of diet on the distribution of carbon isotopes in animals. *Geochim. Cosmochim. Acta* **42**, 495–506 (1978).

97. M. J. Deniro, S. Epstein, Influence of diet on the distribution of nitrogen isotopes in animals. *Geochim. Cosmochim. Acta* **45**, 341–351 (1981).

98. B. N. Smith, S. Epstein, Two categories of c/c ratios for higher plants. *Plant Physiol.* **47**, 380–384 (1971).

99. M. H. O'Leary, Carbon isotope fractionation in plants. *Phytochemistry* **20**, 553–567 (1981).

100. M. Minagawa, E. Wada, Stepwise enrichment of ^{15}N along food chains: Further evidence and the relation between $\delta^{15}N$ and animal age. *Geochim. Cosmochim. Acta* **48**, 1135–1140 (1984).

101. H. Bocherens, D. Drucker, Trophic level isotopic enrichment of carbon and nitrogen in bone collagen: Case studies from recent and ancient terrestrial ecosystems. *Int. J. Osteoarchaeol.* **13**, 46–53 (2003).

102. M. J. Schoeninger, M. J. DeNiro, H. Tauber, Stable nitrogen isotope ratios of bone collagen reflect marine and terrestrial components of prehistoric human diet. *Science***220**, 1381–1383 (1983).

103. M. J. Schoeninger, M. J. DeNiro, Nitrogen and carbon isotopic composition of bone collagen from marine and terrestrial animals. *Geochim. Cosmochim. Acta* **48**, 625–639 (1984).

104. J. Sealy, R. Armstrong, C. Schrire, Beyond lifetime averages: Tracing life histories through isotopic analysis of different calcified tissues from archaeological human skeletons. *Antiquity* **69**, 290–300 (1995).

105. J. P. Gage, *Collagen and Dental Matrices* (Wright, 1989).

106. G. E. Fahy, C. Deter, R. Pitfield, J. J. Miszkiewicz, P. Mahoney, Bone deep: Variation in stable isotope ratios and histomorphometric measurements of bone remodelling within adult humans. *J. Archaeol. Sci.* **87**, 10–16 (2017).

107. S. J. AlQahtani, "Atlas of human tooth development and eruption" (Queen Mary University of London, 2008).

108. M. Price, thesis, University of Cambridge (2013).

109. M. J. DeNiro, Postmortem preservation and alteration of in vivo bone collagen isotope ratios in relation to palaeodietary reconstruction. *Nature* **317**, 806–809 (1985).

110. S. H. Ambrose, Preparation and characterization of bone and tooth collagen for isotopic analysis. *J. Archaeol. Sci.* **17**, 431–451 (1990).

111. A. Rose, thesis, University of Cambridge,Cambridge, UK (2020).

112. C. Malone, S. Stoddart, in *The Oxford Handbook of Prehistoric Figurines*, T. Insoll, Ed. (Oxford Univ. Press, 2017).

113. R. B. Parkinson, *The Tale of Sinuhe and Other Ancient Egyptian Poems, 1940-1640 BC* (Oxford Univ. Press, 1998).

114. M. Danesi, *The History of the Kiss!: The Birth of Popular Culture* (Palgrave Macmillan, 2013).

115. W. R. Jankowiak, S. L. Volsche, J. R. Garcia, Is the romantic-sexual kiss a near human universal? *Am. Anthropol.* **117**, 535–539 (2015).

116. H. Ringbauer, J. Novembre, M. Steinrücken, Human parental relatedness through time–Detecting runs of homozygosity in ancient DNA. *bioRxiv* 2020.05.31.126912 (2020).
